# Genetic Liabilities to Neuropsychiatric Conditions in Suicide Deaths With No Prior Suicidality

**DOI:** 10.1001/jamanetworkopen.2025.38204

**Published:** 2025-10-20

**Authors:** Hilary Coon, Andrey A. Shabalin, Eric T. Monson, Emily DiBlasi, Seonggyun Han, Lisa M. Baird, Erin A. Kaufman, Douglas Tharp, Michael J. Staley, Zhe Yu, Qingqin S. Li, Sarah M. Colbert, Amanda V. Bakian, Anna R. Docherty, Andrew M. McIntosh, Heather C. Whalley, Dierdre Amaro, David K. Crockett, Niamh Mullins, Brooks R. Keeshin

**Affiliations:** 1 Department of Psychiatry, Huntsman Mental Health Institute, University of Utah School of Medicine, Salt Lake City; 2 Utah State Office of the Medical Examiner, Utah Department of Health and Human Services, Salt Lake City; 3 Huntsman Cancer Institute, Salt Lake City, Utah; 4 Neuroscience Therapeutic Area, Janssen Research and Development, Titusville, New Jersey; 5 Now with CHDI Management Inc, Princeton, New Jersey; 6 Department of Psychiatry, Icahn School of Medicine at Mount Sinai, New York, New York; 7 Department of Genetics and Genomic Sciences, Icahn School of Medicine at Mount Sinai, New York, New York; 8 Charles Bronfman Institute for Personalized Medicine, Icahn School of Medicine at Mount Sinai, New York, New York; 9 Division of Psychiatry, Centre for Clinical Brain Sciences, The University of Edinburgh, Edinburgh, Scotland; 10 Generation Scotland, Centre for Medical Informatics, Usher Institute, The University of Edinburgh, Edinburgh, Scotland; 11 Clinical Analytics, Intermountain Health, Salt Lake City, Utah; 12 Department of Pediatrics, University of Utah, Salt Lake City; 13 Primary Children’s Hospital Center for Safe and Healthy Families, Salt Lake City, Utah; 14 Department of Public Health and Caring Science, Child Health and Parenting (CHAP), Uppsala University, Uppsala, Sweden

## Abstract

**Importance:**

Although suicide attempt is the most robust estimator of suicide death, few individuals who attempt it go on to die by suicide (<10%), and approximately 50% of suicide deaths occur in the absence of evidence of prior attempts. The risks are particularly poorly understood in this group.

**Objective:**

To study underlying polygenic liabilities among suicide deaths without evidence of prior nonfatal suicidality (SD-N) compared with suicide deaths with prior suicidality (SD-S), testing prior results showing significantly lower clinical risks of neuropsychiatric traits in SD-N vs SD-S.

**Design, Setting, and Participants:**

In this cohort study, polygenic scores (PGS) were computed using summary statistics from 12 published source studies, then compared across SD-N and SD-S groups taken from the Utah Suicide Mortality Research Study (cases accrued between December 1998 and October 2022). PGS from the suicide death cohorts were also compared to unselected population controls. Evidence of prior suicidality was determined from diagnoses and clinical notes.

**Main Outcomes and Measures:**

Cohort differences in PGS reflecting neuropsychiatric conditions were tested using analysis of covariance, adjusting for sex, age, and genetic ancestry, followed by additional analyses within sex and within subgroups defined by age at death (50 years or younger vs older than 50 years). PGS spanned 12 neuropsychiatric conditions. Data were analyzed between July 2024 and July 2025.

**Results:**

The SD-N cohort (n = 1337) had significantly more male suicide deaths (1105 [82.65%] vs 974 [67.95%]), with an older mean (SD) age at death (47.5 [18.9] vs 41.4 [15.6] years) than the SD-S cohort (n = 1432). The control cohort (n = 19 499) had significantly fewer males (8597 [44.09%]) than both suicide death subsets. Genetic ancestry was similar across the SD-N and SD-S groups (96.77% and 96.81% European ancestry), and control (97.38% European ancestry) groups. Socioeconomic status was not significantly different across suicide cohorts adjusted for age and sex (occupation ranking SD-N mean [SD], 57.16 [24.54]; SD-S mean [SD], 54.72 [25.29]; *t* = 1.30; *P* = .70; maximum education SD-N mean [SD], 2.70 [1.12]; SD-S mean [SD], 2.67 [1.13]; Fisher exact test *P* = .38). Comparing SD-N to SD-S revealed significantly lower (false discovery rate *P* < .05) PGS in the SD-N group for major depressive disorder (adjusted mean difference, 0.085 [95% CI, 0.018-0.152]; *P* = .01), depressed affect (adjusted mean difference, 0.081 [95% CI, 0.012-0.149]; *P* = .02), anxiety (adjusted mean difference, 0.091 [95% CI, 0.021-0.161]; *P* = .01), neuroticism (adjusted mean difference, 0.102 [95% CI, 0.033-0.171]; *P* = .004), and Alzheimer disease (adjusted mean difference, 0.090 [95% CI, 0.021-0.1658]; *P* = .01), and lower (false discovery rate *P* < .10) PGS in SD-N for posttraumatic stress disorder (adjusted mean difference, 0.070 [95% CI, 0.001-0.139]; *P* = .04). Of note, SD-N PGS were not significantly different from controls for depressed affect (adjusted mean difference, 0.037 [95% CI, −0.019 to 0.093]), neuroticism (adjusted mean difference, −0.001 [95% CI, −0.057 to 0.055]), or Alzheimer disease (adjusted mean difference, −0.027 [95% CI, −0.083 to 0.029]).

**Conclusions and Relevance:**

In this cohort study, SD-N showed significantly different genetic liabilities to neuropsychiatric conditions from SD-S. Results have implications for future suicide research and prevention for persons at risk of mortality.

## Key Points

**Question:**

Do underlying genetic liabilities associated with neuropsychiatric conditions in suicide deaths without evidence of prior suicidality differ from liabilities in suicide deaths with prior suicidality?

**Findings:**

This cohort study involving 2769 suicide deaths found that persons in the cohort without prior suicidality showed significantly lower polygenic liabilities associated with neuropsychiatric conditions, mirroring previous clinical results.

**Meaning:**

Differences in underlying genetic liabilities in suicide deaths without prior suicidality suggest a departure from the traditional assumption that neuropsychiatric traits form a sole central core of suicide mortality risk and impel new directions for future research and prevention efforts.

## Teaser

This cohort study compares polygenic liabilities associated with neuropsychiatric conditions between suicide deaths with vs without evidence of prior nonfatal suicidality.

## Introduction

Suicide death is a significant public health crisis, with 49 449 suicide deaths reported in 2022 in the US^1^ and more than 700 000 per year worldwide.^2^ Risks specific to suicide mortality remain largely elusive.^3,4^ The best estimator of suicide mortality is a prior attempt, but only 2.5% to 7.0% of individuals who make suicide attempts go on to die by suicide.^5,6,7^ Additionally, half of persons who die by suicide have no evidence of prior suicidal thoughts or behaviors (suicidality),^8,9,10^ and half show no evidence of psychiatric diagnoses,^11,12,13^ suggesting that while attempts and psychopathology are important, they are insufficient estimators of suicide death.

**Figure 1.**
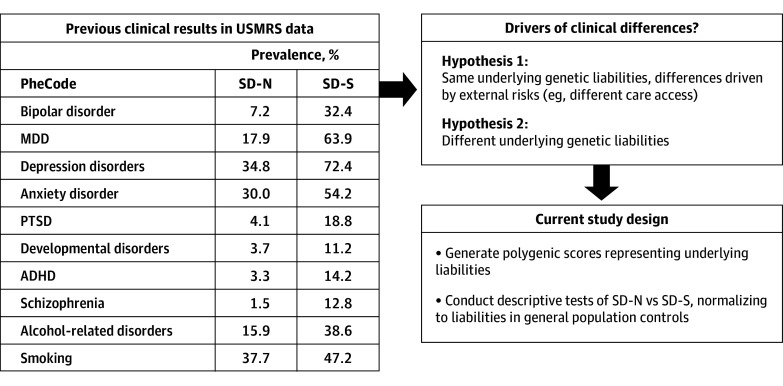
Design of the Current Study Previous clinical results in the Utah Suicide Mortality Research Study (USMRS) cohort.^14^ Results were adjusted for age, sex, number of diagnoses; aggregated clinical categories were defined using PheCodeMap 1.2.^15^ ADHD represents attention-deficit/hyperactivity disorder; MDD, major depressive disorder; PTSD, posttraumatic stress disorder; SD-N, suicide deaths without evidence of prior nonfatal suicidality; SD-S, suicide deaths with prior suicidality.

Our current study (Figure 1) stems from prior Utah Suicide Mortality Research Study (USMRS)^14^ efforts showing significantly fewer neuropsychiatric diagnoses among persons who died by suicide without prior nonfatal suicidality (SD-N) compared with persons who died by suicide with prior suicidality (SD-S) (eTables 1-3 in Supplement 1).^15^ Such results could be attributed to external factors (eg, poor mental health screening or access) or to differences in underlying genetic liabilities. USMRS data resources can be used to address this knowledge gap, testing the hypothesis that underlying genetic neuropsychiatric liabilities may differ between SD-S and SD-N, by using polygenic scores (PGS) as proxies for underlying risks of 12 neuropsychiatric conditions. We calculated the PGS using summary statistics from recent studies selected for size and for matching of ascertainment and genetic ancestry to the USMRS resource using a preregistered study design.^16^ Published source studies for PGS were chosen for large sample size, population ascertainment (when possible), and ancestry matching, and ranged from severe psychopathology to more general symptomatology, and from neurodevelopmental to adult-onset conditions. Although not significant in the clinical study,^14^ Alzheimer disease was added to the present study to assess for possible cross-cutting underlying genetic risks in the neurodegenerative domain.

## Methods

### Suicide Death Sample

This study is a subset of 2769 suicide deaths from the USMRS sample with electronic health records, clinical notes for natural language processing (NLP), and genome-wide genotyping data. This subset represents 17.59% of the deaths in the study collection period, from December 1998 through October 2022 (eTable 4 in Supplement 1). The USMRS resource has been described in detail elsewhere.^17,18^ Whole blood was collected from persons who died by suicide when suicide determination was made by the Utah State Office of the Medical Examiner following detailed investigation. When sufficient sample amounts were available, high-quality DNA was extracted. Identifiers from suicide deaths were securely transferred to staff at the Utah Population Database, a state-wide database of more than 11 million individuals.^19^ The staff linked health records and supplied clinical notes to a suicidality-detecting NLP algorithm.^14^ For this study, identifiers were stripped, rare diagnoses and demographic groups were aggregated, and NLP results were collapsed into positive or negative suicidality before data were given to the research team. This study was approved and consent was waived by the University of Utah, Intermountain Health, and Utah Department of Health and Human Services institutional review boards due to the use of deidentified data on deceased individuals. Reporting of this study followed the Strengthening the Reporting of Observational Studies in Epidemiology (STROBE) reporting guideline.

### Population Controls

Suicide cohort comparisons were normalized to control genotype data from population cohorts similar to the Utah suicide cohort regarding genetic ancestry, including individuals (n = 20 368) from the UK10K^20^ and Generation Scotland^21^ resources. Effects of relatedness were minimized by using only founders in the Generation Scotland resource and randomly deleting 1 in each of 869 related pairs of controls (<third degree) in UK10K. For within-sex analyses, related individuals of the opposite sex were removed to preserve sample size. Within-control analyses were conducted to eliminate single-nucleotide variants (SNVs) with substantial differences across control groups. Molecular data from these controls represent broad population ascertainment, although the UK10K resource includes some additional cohorts with common and rare conditions.^20^ Because available control data consisted only of genetic information, assessment or exclusion of suicidality was not possible. This control resource contained population rates of suicidality^22^ providing a comparison with an unselected sample.

### Demographic and Clinical Data on Suicide Deaths

Demographic and clinical USMRS data included age at death, sex, marital status, maximum educational level (1, some high school; 2, high school diploma; 3, some college; 4, college degree; 5, post college), ranking of occupational status,^23^ and clinical notes and diagnoses from electronic health records. Suicide deaths were categorized as having evidence of prior suicidality based on diagnostic data through *International Classification of Diseases, Ninth or Tenth Revision* diagnostic codes and augmented with additional evidence of prior suicidality as detected through NLP, which increased the number of SD-Ss by 32.50% above diagnostic codes alone^14^ (eTables 5-10 in Supplement 1).^14,24^

### Genotyping of Suicide Deaths

Genotyping was conducted using the Illumina Infinium PsychArray^25^; quality control was performed using GenomeStudio.^26^ Genetic ancestry was estimated with a modification of the kgp2anc algorithm^27^ using 1000 Genomes data^28^ as a reference. Autosomal SNVs were retained if the GenTrain score was higher than 0.5 and the cluster separation score was higher than 0.4. SNVs with more than 5% missing genotypes were removed; samples with a SNV call rate of less than 95% and European ancestry less than 80% were removed per our preregistered design. The SNVs assayed in both suicide and control cohorts were combined, restricted to those only occurring across all cohorts, and then jointly imputed (eAppendix in Supplement 1). For 62 pairs of related individuals (lower than third degree), 1 relative was randomly omitted. However, for within-sex analyses, related individuals of the opposite sex were preferentially removed when possible in order to preserve as many cases as possible in these smaller subsets.

### Computation of PGS

The PGS were computed for the SD-N (n = 1337), SD-S (n = 1432), and control (n = 19 499) cohorts using summary statistics from 12 neuropsychiatric studies (eTable 11 in Supplement 1), with source sample sizes ranging from 31 880 to 1 126 563. These studies were chosen to match the USMRS regarding ancestry (primarily European) and ascertainment (population cohorts) and preregistered as part of our study design.^16^ The PGS of height^29^ was also included as a neutral variable to assess ancestry stratification. PRSice version 2.0^30^ was used to calculate individual PGS as specified in our preregistered design.^16^ PGS are weighted quantitative summary scores reflecting genetic trait liability rather than clinical presence of that trait. A score for an individual is the summation of dosage of minor alleles for each SNV multiplied by the effect size of that allele at that SNV in the discovery genome-wide association study. Our analysis was inclusive of all SNVs in the source studies (threshold *P* value > .99). PGS in this study were descriptive and broadly associated with underlying polygenic liabilities.^31^ PGS calculated with PRS-CS,^32^ a method using a bayesian regression framework and SNV filtering, were used to assess whether the results were robust to the choice of PGS method.

### Statistical Analysis

Analysis of covariance was used to compare the SD-N cohort with the SD-S cohort and each subset to the control cohort, adjusting for sex, age at death, and 20 genetic ancestry principal components, and controlling within-cohort tests for multiple testing using the Benjamini-Hochberg false discovery method.^33^ For ease of interpretation, PGS from the suicide mortality subsets for each PGS outcome were normalized to control (control mean [SD] PGS, 0 [1]). Results unadjusted for sex and age at death allowed for inspection of these moderating factors. Sensitivity analyses to ancestry threshold and PGS method were also conducted.

Because of SD-N vs SD-S cohort sex differences and because neuropsychiatric conditions exhibit strong sex differences,^34^ tests were stratified by sex. Female suicide sample sizes were 238 in the SD-N cohort, 469 in the SD-S cohort, and 10 983 in the control cohort. Male suicide sample sizes were 1113 in the SD-N cohort, 979 in the SD-S cohort, and 8670 in the control cohort. Within-sex analyses were performed as described in the Statistical Analysis section. Because of additional strong SD-N vs SD-S cohort differences in age at death, tests were also conducted within age cohorts (≤50 years, 713 in SD-N and 984 in SD-S; and >50 years, 546 in SD-N and 406 in SD-S). This threshold was determined comparing age at death distributions for the SD-N and SD-S cohorts (eFigure in Supplement 1).

All analyses were conducted from July 2024 to July 2025 using SAS, version 9.4 (SAS Institute Inc) and R, version 4.5.0 (R Project for Statistical Computing). A 2-sided value of *P* < .05 was considered nominally statistically significant. Results were corrected within cohort tests for multiple comparisons using the Benjamini-Hochberg false discovery method,^33^ and results with false discovery rate (FDR) *P* < .05 and FDR *P* < .10 are reported.

## Results

The SD-N cohort (1337; 232 [17.35%] female and 1105 [82.65%] male) compared with the SD-S cohort (1432; 459 [32.05%] female and 973 [67.95%] male) had significantly more male deaths (*P* < .001) and older mean (SD) age at death (47.5 [18.9] vs 41.4 [15.6] years; *P* < .001). The 2 suicide death subtypes were not significantly different regarding genetic ancestry; both were predominantly European (96.77% vs 96.81%; *P* = .72). Matching population expectations,^1,2^ controls (n = 19 499; 97.38% European ancestry) included significantly more females (10 902 [55.91%]; male, 8579 [44.09%]; *P* < .001) than either of the suicide death cohorts. Adjusting for age and sex, the SD-N and SD-S cohorts were not significantly different from occupation ranking (mean [SD] SD-N, 57.16 [24.54]; mean [SD] SD-S, 54.72 [25.29]; *t* = 1.30; *P* = .70), maximum educational level (mean [SD] SD-N, 2.70 [1.12]; mean [SD] SD-S, 2.65 [1.13]; Fisher exact test *P* = .38), or likelihood to ever be married (SD-N, 830 of 1169 [71.0%]; SD-S, 789 of 1257 [62.77%]; χ^2^ = 1.23; *P* = .27), although persons in the SD-S cohort were more likely than those in the SD-N cohort to be divorced (SD-N, 302 of 783 [38.57%]; SD-S, 351 of 751 [46.74%]; χ^2^ = 6.20; *P* = .01).

### PGS Results

PGS across the 12 traits were relatively independent (eTable 12 in Supplement 1). Of 66 possible correlations, most (n = 60) were low (*r*, 0.00-0.19); 5 were modest (*r*, 0.20-0.33), and 1 (neuroticism vs depressed affect) was strong (*r*, 0.78).

**Figure 2.**
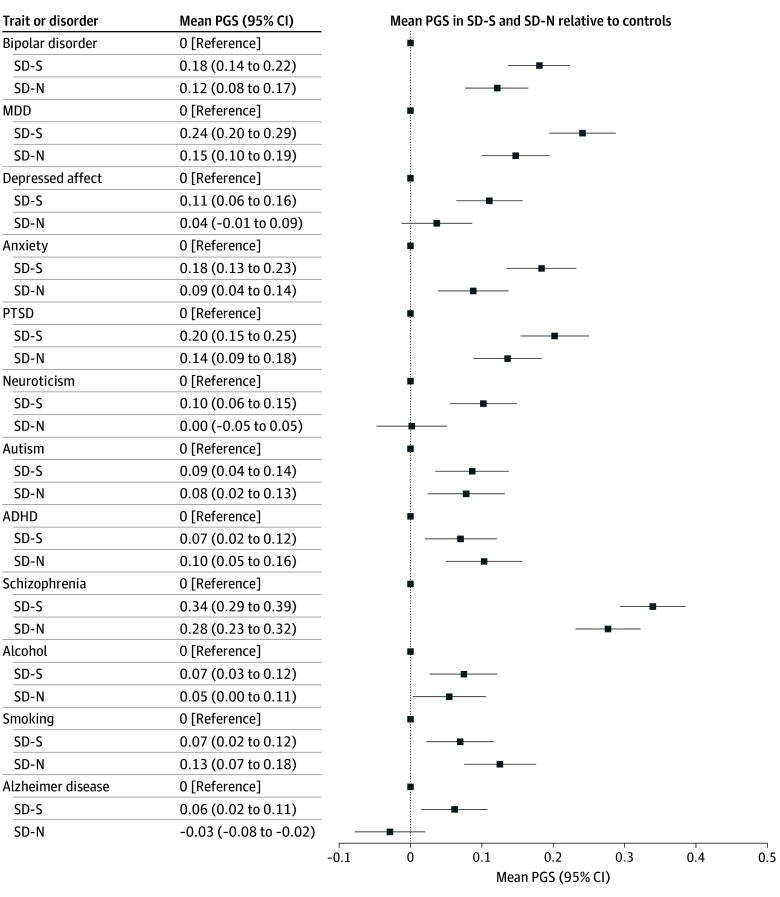
Polygenic Score (PGS) Analysis of Covariance Results for Suicide Mortality Cohorts Results for suicide subtypes are normalized to the controls, which are represented by the zero line in the graph. All tests were adjusted for sex, age at death (for suicide cohorts), and 20 genetic ancestry principal components. ADHD represents attention-deficit/hyperactivity disorder; MDD, major depressive disorder; PTSD, posttraumatic stress disorder; SD-N, suicide deaths without evidence of prior nonfatal suicidality; SD-S, suicide deaths with prior suicidality.

Table and Figure 2 show SD-N vs SD-S subtype differences. For ease of interpretation and assessment of effect sizes, all results were normalized to the control group (control mean [SD], 0 [1]). Results indicated significantly lower (FDR *P* < .05) PGS in the SD-N vs SD-S subtypes for major depressive disorder (MDD; adjusted mean difference, 0.085 [95% CI, 0.018-0.152]; *P* = .01), depressed affect (adjusted mean difference, 0.081 [95% CI, 0.012-0.149]; *P* = .02), anxiety (adjusted mean difference, 0.091 [95% CI, 0.021-0.161]; *P* = .01), neuroticism (adjusted mean difference, 0.102 [95% CI, 0.033-0.171]; *P* = .004), and Alzheimer disease (AD; adjusted mean difference, 0.090 [95% CI, 0.021-0.1658]; *P* = .01), and lower (FDR *P* < .10) in the SD-N subtype than in the SD-S subtype for posttraumatic stress disorder (PTSD; adjusted mean difference, 0.070 [95% CI, 0.001-0.139]; *P* = .04). Both bipolar disorder (adjusted mean difference, 0.059 [95% CI, −0.004 to 0.121]; *P* = .06) and schizophrenia (adjusted mean difference, 0.061 [95% CI, −0.004 to 0.126]; *P* = .07) showed lower PGS in the SD-N vs SD-S subtypes at an FDR of 10% (FDR *P* = .10). Percentages of variance explained by PGS were small (<1%). Tests unadjusted for sex or age at death (eTable 13 in Supplement 1) showed similar results, although as expected, the statistical significance of the SD-N vs SD-S subtypes was increased (adjusted mean difference range, 0.080-0.113; *P* value range, .02 to <.001), and PGS were significantly lower in the SD-N subtype for schizophrenia (FDR *P* < .05; adjusted mean difference, 0.075 [95% CI, 0.010-0.139]; *P* = .02).

**Table.** PGS Analysis of Covariance Results Comparing Suicide Death Cohorts^a^

Trait or diagnosis	PGS, mean (SE)^b^		SD-N vs SD-S adjusted mean difference (95% CI)	*P* value	FDR-adj *P* value^c^	SD-N vs control adjusted mean difference (95% CI)	*P* value	FDR-adj *P* value^c^	SD-S vs control adjusted mean difference (95% CI)	*P* value	FDR-adj *P* value^c^
	SD-N	SD-S									
Bipolar disorder	0.121 (0.022)	0.181 (0.022)	0.059 (−0.004 to 0.121)	.06	.10	0.127 (0.071 to 0.183)	<.001	<.001	0.186 (0.133 to 0.238)	<.001	<.001
MDD	0.147 (0.024)	0.241 (0.024)	0.085 (0.018 to 0.152)	.01	.04	0.158 (0.102 to 0.2143)	<.001	<.001	0.249 (0.195 to 0.302)	<.001	<.001
Depressed affect	0.0367 (0.025)	0.111 (0.024)	0.081 (0.012 to 0.149)	.02	.05	0.037 (−0.019 to 0.093)	.20	.23	0.111 (0.057 to 0.164)	<.001	<.001
Anxiety	0.088 (0.025)	0.184 (0.025)	0.091 (0.021 to 0.161)	.01	.04	0.087 (0.031 to 0.143)	.002	.004	0.182 (0.129 to 0.236)	<.001	<.001
PTSD	0.136 (0.024)	0.202 (0.024)	0.070 (0.001 to 0.139)	.04	.09	0.121 (0.065 to 0.177)	<.001	<.001	0.191 (0.137 to 0.245)	<.001	<.001
Neuroticism	0.002 (0.025)	0.102 (0.024)	0.102 (0.033 to 0.171)	.004	.04	−0.001 (−0.057 to 0.055)	.97	.97	0.101 (0.048 to 0.155)	<.001	<.001
Autism	0.078 (0.027)	0.086 (0.026)	0.005 (−0.070 to 0.080)	.89	.89	0.080 (0.023 to 0.136)	.006	.008	0.085 (0.031 to 0.139)	.002	.003
ADHD	0.103 (0.027)	0.070 (0.026)	−0.038 (−0.112 to 0.036)	.32	.38	0.112 (0.055 to 0.168)	<.001	<.001	0.076 (0.022 to 0.129)	.006	.007
Schizophrenia	0.277 (0.023)	0340 (0.023)	0.061 (−0.004-0.126)	.07	.10	0.266 (0.210 to 0.322)	<.001	<.001	0.333 (0.280 to 0.387)	<.001	<.001
Alcohol (drinks/wk)	0.055 (0.026)	0.075 (0.024)	0.019 (−0.051 to 0.089)	.60	.65	0.067 (0.011 to 0.123)	.02	.03	0.082 (0.028 to 0.135)	.003	.004
Smoking (ever)	0.125 (.026)	0.070 (0.024)	−0.060 (−0.130 to 0.010)	.09	.12	0.128 (0.071 to 0.184)	<.001	<.001	0.072 (0.018 to 0.126)	.009	.01
Alzheimer disease	−0.029 (0.025)	0.062 (0.024)	0.090 (0.021 to 0.1658	.01	.04	−0.027 (−0.083 to 0.029)	.34	.37	0.064 (0.010 to 0.118)	.02	.02
Height (neutral control PGS)^d^	−0.040 (0.025)	0.011 (0.025)	0.047 (−0.024 to 0.117)	.20	NA	−0.043 (−0.099 to 0.013)	.13	NA	0.009 (−0.045 to 0.063)	.74	NA

Abbreviations: ADHD, attention-deficit/hyperactivity disorder; FDR-adj, adjusted for false discovery rate; MDD, major depressive disorder; NA, not applicable; PGS, polygenic scores; PTSD, posttraumatic stress disorder; SD-N, suicide deaths with no evidence of prior nonfatal suicidality; SD-S, suicide deaths with evidence of prior nonfatal suicidality.

^a^ All tests were adjusted for sex, age at death (in suicide cohorts), and 20 genetic ancestry principal components; incremental variance explained (*R*^2^) by PGS controlling for covariates ranged from 0.0001% to 0.70%.

^b^ All PGS were normalized to controls, giving the controls a mean PGS of 0 and an SD of 1.

^c^ FDR was calculated within each cohort test (SD-N vs SD-S; SD-N vs control; SD-S vs control).

^d^ Results for height, which was included as a check for proper ancestry adjustment, were not included in the FDR correction for substantive results.

Comparisons of the SD-N subtype with the control group showed that SD-N PGS for depressed affect (adjusted mean difference, 0.037 [95% CI, −0.019 to 0.093]), neuroticism (adjusted mean difference, −0.001 [95% CI, −0.057 to 0.055]), and AD (adjusted mean difference, −0.027 [95% CI, −0.083 to 0.029]) were indistinguishable from those of controls. In addition, although scores for autism (adjusted mean difference, 0.005 [95% CI, −0.070 to 0.080]), attention-deficit/hyperactivity disorder (ADHD; adjusted mean difference, −0.038 [95% CI, −0.112 to 0.036]), and alcohol (adjusted mean difference, 0.019 [95% CI, −0.051 to 0.089]) were not significantly different between the SD-N and SD-S subtypes, they showed significant (FDR *P* < .05) and similar increases over the control group PGS (adjusted mean differences, 0.080 [95% CI, 0.023-0.136] and 0.085 [95% CI, 0.031-0.139] for autism; 0.112 [95% CI, 0.055-0.168] and 0.076 [95% CI, 0.022-0.129] for ADHD, and 0.067 [95% CI, 0.011-0.123] and 0.082 [95% CI, 0.028-0.135] for alcohol).

### Sex-Specific Tests

**Figure 3.**
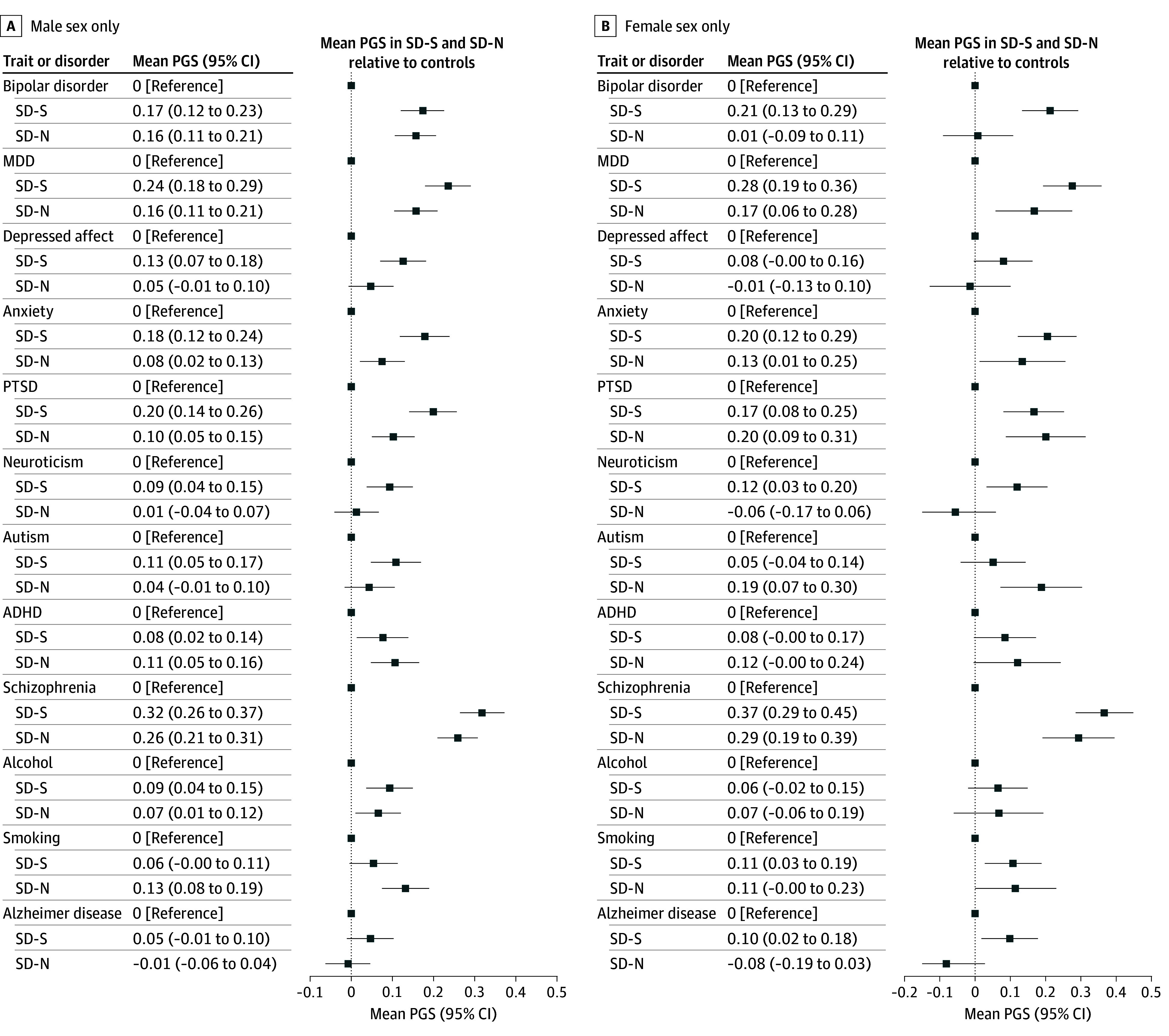
Polygenic Score (PGS) Analysis of Covariance Results for Suicide Mortality Subcohorts Results for suicide subtypes are normalized to the controls, which are represented by the zero line in each panel. All tests were adjusted for age at death (for suicide cohorts) and 20 genetic ancestry principal components; age at death subgroups were also adjusted for sex. ADHD represents attention-deficit/hyperactivity disorder; MDD, major depressive disorder; PTSD, posttraumatic stress disorder; SD-N, suicide deaths without evidence of prior nonfatal suicidality; SD-S, suicide deaths with prior suicidality.

For sex, females (mean [SD], 44.7 [15.1] vs 41.4 [14.6] years; *P* = 004) and males (mean [SD], 48.0 [19.5] vs 41.3 [16.1] years; *P* < .001) in the SD-N subtype died at an older age than in the SD-S subtype. Results in eTable 14 in Supplement 1 and Figure 3 were adjusted for age at death and normalized to control. Results showed lower PGS (FDR *P* < .10) in male SD-N vs male SD-S for anxiety (adjusted mean difference, 0.102 [95% CI, 0.023-0.182]; *P* = .01) and PTSD (adjusted mean difference, 0.097 [95% CI, 0.020-0.173]; *P* = .01). In contrast, PGS were significantly lower (FDR *P* < .05) in female SD-N vs SD-S subtypes for bipolar disorder (adjusted mean difference, 0.204 [95% CI, 0.073-0.335]; *P* = .002), and lower (FDR *P* < .10) for neuroticism (adjusted mean difference, 0.174 [95% CI, 0.029-0.319; *P* = .02) and AD (adjusted mean difference, 0.180 [95% CI, 0.044-0.316]; *P* = .01). Comparisons with controls highlighted additional interesting differences. The male PGS for smoking were increased over those of controls only in the SD-N subtype (adjusted mean difference, 0.132 [95% CI, 0.070-0.194]), and the male PGS for autism were increased over those of controls only in the SD-S subtype (adjusted mean difference, 0.109 [95% CI, 0.043-0.174]). However, among female deaths, the PGS for autism were increased over those of controls only for the SD-N subtype (adjusted mean difference, 0.188 [95% CI, 0.060-0.316]), and the smoking PGS were increased over those of controls only in the SD-S cohort (adjusted mean difference, 0.108 [95% CI, 0.016-0.200]), opposite to these results for male deaths.

### Tests Within Subgroups by Age at Death

**Figure 4.**
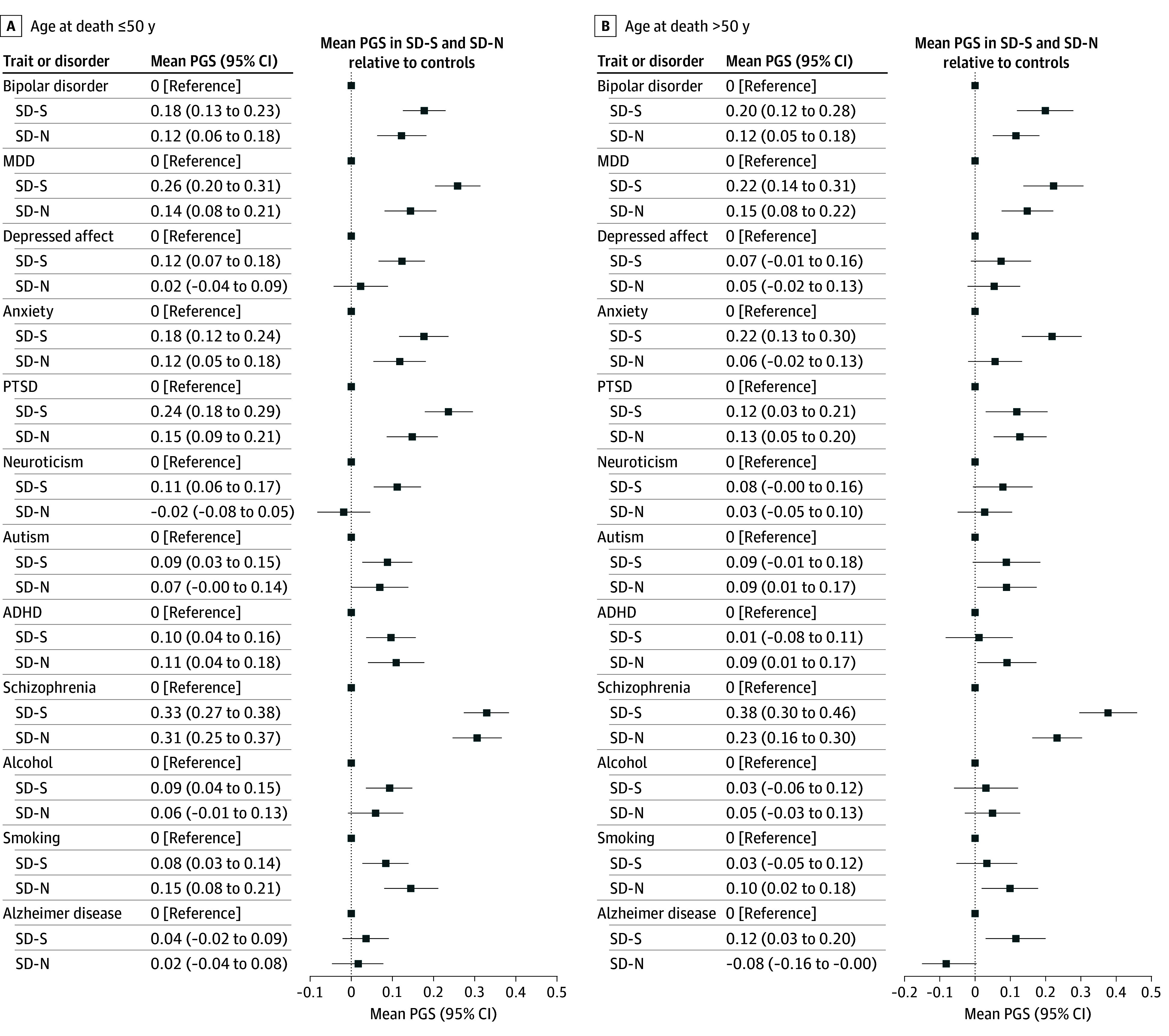
Polygenic Score (PGS) Analysis of Covariance Results for Suicide Mortality Subcohorts Results for suicide subtypes are normalized to the controls, which are represented by the zero line in each panel. All tests were adjusted for age at death (for suicide cohorts) and 20 genetic ancestry principal components; age at death subgroups were also adjusted for sex. ADHD represents attention-deficit/hyperactivity disorder; MDD, major depressive disorder; PTSD, posttraumatic stress disorder; SD-N, suicide deaths without evidence of prior nonfatal suicidality; SD-S, suicide deaths with prior suicidality.

As with the full sample, the SD-N subtype was composed of more male deaths than the SD-S subtype for those aged 50 years or younger (619 of 767 [80.71%] vs 696 of 1026 [67.84%]; *P* < .001) and for those older than 50 years (495 of 581 [85.20%] vs 287 of 420 [68.33%]; *P* < .001). In analyses within the subgroup 50 years or younger at death, PGS in the SD-N cohort were lower (FDR *P* < .10) than in the SD-S cohort for MDD (adjusted mean difference, 0.102 [95% CI, 0.018-0.186]) depressed affect (adjusted mean difference, 0.101 [95% CI, 0.015-0.187]) and neuroticism (adjusted mean difference, 0.121 [95% CI, 0.035-0.208]), and modestly lower for PTSD (adjusted mean difference, 0.092 [95% CI, 0.007-0.178]; *P* = .03) Figure 4. For individuals who were older than 50 years at death, SD-N PGS were lower (FDR *P* < .05) than SD-S PGS for anxiety (adjusted mean difference, 0.179 [95% CI, 0.063-0.295]; *P* = .003) and AD (adjusted mean difference, 0.211 [95% CI, 0.093-0.330]; *P* < .001) and lower (FDR *P* < .10) for schizophrenia (adjusted mean difference, 0.135 [95% CI, 0.026-0.244]; *P* = .02) Figure 4.

In comparisons with controls, again some notable differences were apparent. PGS for anxiety were significantly increased over those of controls only in the subgroup that was 50 years or younger at death (mean difference, 0.118 [95% CI, 0.045-0.190]; *P* = .001) but not older than 50 years at death (adjusted mean difference, 0.054 [95% CI, −0.029 to 0.138]) in the SD-N subtype. While PGS for AD did not differ from those of controls in younger SD-N (adjusted mean difference, 0.018 [95% CI, −0.054 to 0.091]), these PGS were somewhat lower (FDR *P* < .10) than those of controls in older SD-N (adjusted mean difference, −0.080 [95% CI, −0.163 to 0.003]; *P* = .06). Notably, PGS for schizophrenia were significantly and similarly elevated (FDR *P* < .05) over those of controls for both younger SD-N (adjusted mean difference, 0.296 [95% CI, 0.224-0.369]; *P* < .001) and younger SD-S (adjusted mean difference, 0.322 [95% CI, 0.259-0.385]; *P* < .001). However, the schizophrenia PGS difference was increased more statistically significantly in the older SD-S subtype vs that in the control group (adjusted mean difference, 0.371 [95% CI, 0.274-0.467] *P* < .001) than in older SD-N vs controls (adjusted mean difference, 0.221 [95% CI, 0.138-0.304]; *P* < .001).

### Sensitivity Tests

eTables 15 and 16 in Supplement 1 show SD-S, SD-N, and control PGS comparisons restricted to suicides with at least 90% European ancestry and all suicides, respectively. Results with these altered exclusion thresholds showed minor differences in significance but overall consistency of lowered PGS in the SD-N subtype vs the SD-S subtype. eTable 17 in Supplement 1 shows SD-S, SD-N, and control group comparisons for PGS computed using PRS-CS, again showing consistency with the original results.

## Discussion

Prior suicidality is the most robust estimator of suicide death,^5,6,7^ but the association with this estimator is imperfect, and many suicide deaths occur in its absence.^8,9,10^ Previous work using USMRS data^14^ showed that suicides without prior suicidality (SD-N) have significantly fewer neuropsychiatric diagnoses than those with prior suicidality (SD-S). Prior suicide research rests on assumptions that underlying liabilities to neuropsychiatric conditions are ubiquitous risk factors leading to suicide death and would likely attribute these diagnostic differences to less care utilization in SD-N.^35^ However, it is possible that persons who died by suicide without prior suicidality have differences in underlying genetic liabilities to neuropsychiatric conditions. The present study tested this hypothesis with PGS associated with liabilities to neuropsychiatric conditions for 1337 population-ascertained suicide deaths in the SD-N group. These suicide deaths were compared with 1432 suicides with prior suicidality (SD-S), normalized to unselected controls. Evidence of suicidality included diagnostic codes, and any mention of suicidal ideation or behaviors in clinical notes.

Demographic comparisons showed that individuals in the SD-N subgroup were significantly younger and more were male than in the SD-S subgroup. Measures of socioeconomic status (educational level and occupation) showed no significant differences, and SD-N individuals were no more or less likely to be ever married than SD-S individuals, although those in the SD-S subtype were more likely to be divorced. Lower PGS (FDR *P* < .05) for the SD-N vs SD-S subtypes were observed across a spectrum of neuropsychiatric conditions, including MDD, depressed affect, anxiety, neuroticism, and AD, and lower (FDR *P* < .10) for PTSD. In addition, for depressed affect, neuroticism, and AD, the SD-N subtype was not significantly different from the controls. These results indicated a substantially lower degree of underlying genetic liability to many neuropsychiatric conditions for the SD-N subtype. Results were robust to the ancestry exclusion threshold and PGS scoring method.

Our results have implications for future research and clinical interventions, namely, that not all suicide deaths have similar increased underlying liability to neuropsychiatric conditions. For research, combining suicide deaths with evidence and without evidence of prior nonfatal suicidality mixes 2 substantially different genetic subtypes, weakening results specific to suicide mortality. Regarding clinical interventions, a focus solely on mental illness risk reduction may be less effective for individuals for whom this underlying liability may be substantially lower. Further understanding of clinical characteristics, underlying genetic liabilities, and external exposures in SD-N will be required to direct more targeted interventions.

Our analyses also indicated that while many conditions showed lower PGS in SD-N, PGS for ADHD and alcohol were equally increased over those of controls for both suicide subtypes, suggesting underlying shared genetic liabilities associated with characteristics such as poor impulse regulation,^36,37^ regardless of the presence of prior suicidality. PGS for autism were also not lower in SD-N vs SD-S in the combined analysis. However, sex-specific results suggested a potentially more complex picture for these traits. For example, autism PGS were elevated in female (but not male) deaths in the SD-N cohort, suggesting suicide studies involving female sex with neurodevelopmental risks should perhaps address a broader range of characteristics beyond suicidal behaviors or co-occurring psychopathology (eg, cognitive rigidity or social and communication deficits).^38^ Overall, sex differences suggested that male sex may be associated with lower PGS in SD-N for anxiety and PTSD. In contrast, lower PGS in SD-N for bipolar disorder, AD, and neuroticism may be associated with female sex. Results within age at death revealed lower PGS specifically for anxiety and AD among persons who were older at death in the SD-N vs SD-S subtypes, but lower PGS for MDD, depressed affect, and neuroticism among individuals who were younger at death in the SD-N vs SD-S subtypes.

Finally, the schizophrenia PGS showed the most consistent increase over controls of any trait. Interestingly, in prior studies in our data resource, rates of diagnosed schizophrenia in SD-S were high (12.8%), but for SD-N, rates of clinically diagnosed schizophrenia were only modestly increased over individuals in the control group, who had no evidence of lifetime suicidality (1.5%).^14^ The clinical data, therefore, did not indicate high rates of overt schizophrenia in the SD-N subtype. While it is possible that the SD-N subgroup may include individuals with undiagnosed schizophrenia, it is also possible this PGS increase in SD-N represents additional sensitivity of the schizophrenia PGS to detect significant subclinical liability, or that the increase represents other aspects of physical or behavioral traits captured in the schizophrenia PGS^39,40^ that are associated with suicide mortality.

Our subgroup results suggested that pooling PGS across sex and also age at death may mask important signals of risk, especially for associations in opposite directions or with markedly different magnitudes. Subgroup-specific findings suggested there may be underlying genetic liabilities to suicide mortality that differ substantially by sex and age at death, and that these factors are not consistent across different neuropsychiatric outcomes. Future genetic liability risk discovery efforts investigating both psychiatric and nonpsychiatric outcomes leading to suicide mortality should stratify by demographic groups.

### Limitations

This study has limitations. While previous work from members of our team showed the NLP of all clinical notes to identify suicidality greatly improved the capture of individuals in the SD-S group,^14^ some individuals in the SD-N group likely had prior suicidality not represented either in diagnostic codes or the NLP data. However, such individuals would more closely resemble those in the SD-S group, thus creating fewer significant differences between SD-N vs SD-S outcomes. In addition, the population-based controls were unscreened for suicidality, resulting in potential lessening of differences between controls and suicide subsets. The USMRS study, while population-ascertained, includes individuals of predominantly European ancestry, limiting generalizability. The study is confined only to suicide deaths with available data (health records and genetic information). Studies of characteristics of suicide deaths without these critical data sources remain currently out of reach. Sample sizes of each suicide death subset were relatively small, requiring replication and extension with future larger data resources. Small samples were particularly apparent for sex-specific analyses, in which results should be interpreted with caution. Differences by subset suggest sex by age at death analyses may be important, but these analyses will require larger samples. Our study also included only preregistered PGS associated with psychopathology to control for bias. A more general screen of PGS across additional psychiatric and medical conditions in these suicide death subsets is warranted. In addition, it is likely that other demographic stratifications may also reveal important results. Finally, our study did not address mediating or moderating factors of social and environmental exposures, which will be critical to achieve a fuller understanding of mortality risk.

## Conclusions

This cohort study provides important first steps in determining underlying risks in suicide deaths with no evidence of prior nonfatal suicidality, a large subtype previously unavailable for study. Results of this work showed that the previously demonstrated markedly reduced evidence of neuropsychiatric diagnoses in clinical data in this subset^14^ is associated with lower underlying polygenic liabilities associated with neuropsychiatric conditions rather than associated with similar underlying liabilities but a lack of care access. The results also justify additional studies of nonpsychiatric characteristics, social and environmental exposures, and diverse global cohorts, and indicate that future studies within sex, age at death, and perhaps other demographic subsets will be essential. However, pending replication, the findings indicate that individuals with suicide death without evidence of prior nonfatal suicidality, who represent roughly half of persons who died by suicide,^8,9,10^ may have different genetic etiology from persons who died by suicide who do have the robust risk estimator of prior suicidality. These findings have implications for future research and development of future interventions to prevent mortality.

## Notes

Supplement 1. **eTable 1.** Neuropsychiatric clinical results from previous published studies of Utah suicide mortality cohorts with (SD-S) and without (SD-N) prior nonfatal suicidality **eTable 2.** Physical medical clinical results from previous published studies of Utah suicide mortality cohorts with (SD-S) and without (SD-N) prior nonfatal suicidality **eTable 3.** Clinical Phecode results from previous published studies of Utah suicide mortality cohorts with (SD-S) and without (SD-N) prior nonfatal suicidality with protective effects **eTable 4.** Comparisons of basic demographics between suicide deaths with vs without available clinical data and genotyping **eTable 5.** Characteristics of EHR notes in the manual validation of the NLP **eTable 6.** Validation details of the NLP within individual notes by note type (psychiatric vs non-psychiatric encounter) **eTable 7.** Rater agreement details and kappa statistics **eTable 8.** Kappa statistics for rater agreement **eTable 9.** Final metrics from manual validation of the NLP in individual notes **eTable 10.** NLP performance considering the entire corpus of notes for each person as the source text, rather than individual notes **eAppendix.** Additional details of imputation methods **eTable 11.** Source studies for polygenic score statistics used to test for underlying neuropsychiatric genetic vulnerabilities **eTable 12.** Correlations among the PGS scores used in this study **eFigure.** Age at death distributions for SD-N and SD-S **eTable 13.** Polygenic score comparisons without adjustment for the moderating variables of sex and age at death **eTable 14.** Polygenic score (PGS) analysis of covariance results comparing suicide death cohorts within subsets defined by sex and by age at death **eTable 15.** Polygenic score comparisons including suicides with ≥90% European ancestry; combined males and females **eTable 16.** Polygenic score comparisons including all suicides; combined males and females with no ancestry threshold **eTable 17.** Comparisons of PGS derived using PRS-CS (suicides are restricted to 80% European), combined males and females

Supplement 2.
Data Sharing Statement
