# Supplementary material for: Genetic Liabilities to Neuropsychiatric Conditions in Suicide Deaths With No Prior Suicidality
Source: JAMA Netw Open. 2025 Oct 20;8(10):e2538204. doi: 10.1001/jamanetworkopen.2025.38204 (PMC12538367; doi:10.1001/jamanetworkopen.2025.38204)
Supplement: Supplement 1. — eTable 1. Neuropsychiatric clinical results from previous published studies of Utah suicide mortality cohorts with (SD-S) and without (SD-N) prior nonfatal suicidality eTable 2. Physical medical clinical results from previous published studies of Utah suicide mortality cohorts with (SD-S) and without (SD-N) prior nonfatal suicidality eTable 3. Clinical Phecode results from previous published studies of Utah suicide mortality cohorts with (SD-S) and without (SD-N) prior nonfatal suicidality with protective effects eTable 4. Comparisons of basic demographics between suicide deaths with vs without available clinical data and genotyping eTable 5. Characteristics of EHR notes in the manual validation of the NLP eTable 6. Validation details of the NLP within individual notes by note type (psychiatric vs non-psychiatric encounter) eTable 7. Rater agreement details and kappa statistics eTable 8. Kappa statistics for rater agreement eTable 9. Final metrics from manual validation of the NLP in individual notes eTable 10. NLP performance considering the entire corpus of notes for each person as the source text, rather than individual notes eAppendix. Additional details of imputation methods eTable 11. Source studies for polygenic score statistics used to test for underlying neuropsychiatric genetic vulnerabilities eTable 12. Correlations among the PGS scores used in this study eFigure. Age at death distributions for SD-N and SD-S eTable 13. Polygenic score comparisons without adjustment for the moderating variables of sex and age at death eTable 14. Polygenic score (PGS) analysis of covariance results comparing suicide death cohorts within subsets defined by sex and by age at death eTable 15. Polygenic score comparisons including suicides with ≥90% European ancestry; combined males and females eTable 16. Polygenic score comparisons including all suicides; combined males and females with no ancestry threshold eTable 17. Comparisons of PGS derived using PRS-CS (suicides are rest [file jamanetwopen-e2538204-s001.pdf]

Supplementary Online Content

Coon H, Shabalin AA, Monson ET, et al. Genetic liabilities to neuropsychiatric conditions in suicide deaths with no prior suicidality. *JAMA Netw Open*. 2025;8(10):e2538204. doi:10.1001/jamanetworkopen.2025.38204

eTable 1. Neuropsychiatric clinical results from previous published studies of Utah suicide mortality cohorts with (SD-S) and without (SD-N) prior nonfatal suicidality

eTable 2. Physical medical clinical results from previous published studies of Utah suicide mortality cohorts with (SD-S) and without (SD-N) prior nonfatal suicidality

eTable 3. Clinical Phecode results from previous published studies of Utah suicide mortality cohorts with (SD-S) and without (SD-N) prior nonfatal suicidality with protective effects

eTable 4. Comparisons of basic demographics between suicide deaths with vs. without available clinical data and genotyping

eTable 5. Characteristics of EHR notes in the manual validation of the NLP

eTable 6. Validation details of the NLP within individual notes by note type (psychiatric vs. non-psychiatric encounter)

eTable 7. Rater agreement details and kappa statistics

eTable 8. Kappa statistics for rater agreement

eTable 9. Final metrics from manual validation of the NLP in individual notes

eTable 10. NLP performance considering the entire corpus of notes for each person as the source text, rather than individual notes

eAppendix. Additional details of imputation methods

eTable 11. Source studies for polygenic score statistics used to test for underlying neuropsychiatric genetic vulnerabilities

eTable 12. Correlations among the PGS scores used in this study

eFigure. Age at death distributions for SD-N and SD-S

eTable 13. Polygenic score comparisons without adjustment for the moderating variables of sex and age at death

© 2025 Coon H et al. *JAMA Network Open.*

eTable 14. Polygenic score (PGS) analysis of covariance results comparing suicide death cohorts within subsets defined by sex and by age at death

eTable 15. Polygenic score comparisons including suicides with ≥90% European ancestry; combined males and females

eTable 16. Polygenic score comparisons including all suicides; combined males and females with no ancestry threshold

eTable 17. Comparisons of PGS derived using PRS-CS (suicides are restricted to 80% European), combined males and females

This supplementary material has been provided by the authors to give readers additional information about their work.

© 2025 Coon H et al. *JAMA Network Open*.

eTable 1. Neuropsychiatric clinical results from previous published studies of Utah suicide mortality cohorts with (SD-S) and without (SD-N) prior nonfatal suicidality (SI/SB; Coon et al., 2025).[15]

Logistic regressions were performed for presence/ absence of neuropsychiatric PheCodes to determine effects of suicide death vs. control status (suicide death effect, independent of effects of presence of nonfatal suicidality) and also on presence/absence of suicidality (presence of SI/SB effect, independent of suicide mortality status). **The table shows neuropsychiatric conditions are far more strongly associated with presence of nonfatal suicidality than with actual death by suicide, with the exception of the final two substance use disorder (SUD) Phecodes.**

PheCode	Grouping	Odds ratio: suicide death (p-value)	Odds ratio: presence SI/SB (p-value)
Depression	Affective	**2.56 (8.32E-85)**	**8.45 (<E-350)**
Bipolar	Affective	**2.21 (3.31E-42)**	**8.32 (1.47E-283)**
Major Depressive Disorder	Affective	**1.86 (3.34E-35)**	**11.07 (<E-350)**
Dysthymic disorders	Affective	**1.56 (3.50E-11)**	**3.50 (6.96E-81)**
Other mood disorders	Affective	**1.44 (4.76E-08)**	**7.44 (2.26E-193)**
Somatoform disorder	Anxiety/stress	**2.38 (9.54E-14)**	**2.43 (1.96E-12)**
Anxiety-related states & conditions	Anxiety/stress	**2.20 (7.03E-35)**	**14.88 (8.18E-304)**
Posttraumatic stress disorder	Anxiety/stress	**2.09 (5.29E-24)**	**6.78 (9.40E-132)**
Anxiety disorder	Anxiety/stress	**2.03 (1.78E-52)**	**4.01 (1.13E-265)**
Agoraphobia, social phobia, panic disorder	Anxiety/stress	**1.70 (3.76E-16)**	**4.57 (1.50E-122)**
Obsessive-compulsive disorders	Anxiety/stress	**1.50 (8.77E-05)**	**5.48 (2.11E-53)**
Generalized anxiety disorder	Anxiety/stress	**1.43 (4.08E-10)**	**5.01 (2.34E-203)**
Eating disorders	Anxiety/stress	1.62 (0.015)	**4.56 (3.27E-11)**
Acute reaction to stress	Anxiety/stress	1.31 (0.0069)	**2.13 (4.20E-15)**
Adjustment reaction	Anxiety/stress	1.28 (0.0027)	**3.43 (1.77E-54)**
Personality disorders (excluding antisocial PD and borderline PD)	PD	**2.61 (2.72E-28)**	**10.61 (5.20E-109)**
Antisocial PD & borderline PD	PD	**2.42 (8.66E-23)**	**11.10 (2.29E-101**
Conduct disorders	Impulsive	1.98 (0.072)	**5.56 (6.02E-65)**
Attention deficit hyperactivity disorder	Impulsive	1.97 (0.0067)	**3.18 (2.59E-81)**
Developmental delays & disorders	Developmental	1.08 (0.301)	**2.69 (7.70E-56)**
Delirium	Psychotic	**2.37 (1.10E-18)**	**2.75 (8.77E-23)**
Alteration of consciousness	Psychotic	**2.18 (2.55E-30)**	**2.42 (9.57E-39)**
Paranoid disorders	Psychotic	**2.08 (5.77E-13)**	**8.90 (4.87E-73)**
Psychosis	Psychotic	**1.98 (1.77E-23)**	**6.18 (5.65E-142)**
Schizophrenia	Psychotic	**1.90 (9.66E-15)**	**10.37 (1.15E-134)**
Altered mental status	Psychotic	**1.86 (2.88E-17)**	**2.43 (2.19E-35)**
Hallucinations	Psychotic	1.46 (0.0014)	**7.24 (3.89E-48)**
Substance addiction & disorders	SUD	**3.00 (1.95E-113)**	**5.18 (2.51E-308)**
Alcoholism	SUD	**2.77 (6.01E-94)**	**4.16 (1.96E-197)**
Alcohol-related disorders	SUD	**2.75 (4.06E-68)**	**5.23 (2.13E-170)**
Adverse events of opiates & narcotics in therapeutic use	SUD	**2.10 (2.54E-24)**	**2.02 (7.75E-23)**
Tobacco use disorder	SUD	**2.05 (6.96E-67)**	**2.10 (6.97E-85)**

Notes. Odds ratios were derived from logistic regressions. Each binary presence/absence PheCode was regressed on case-control status (suicide death effect) and then on presence/absence of SI/SB (presence SI/SB effect), adjusting for age, sex, and transformed overall number of PheCodes. Tests that were significant after adjustment for multiple testing are shown in bold type.

© 2025 Coon H et al. *JAMA Network Open*.

eTable 2. Physical medical clinical results from previous published studies of Utah suicide mortality cohorts with (SD-S) and without (SD-N) prior nonfatal suicidality (SI/SB; Coon et al., 2025).[15]

Logistic regressions were performed for presence/ absence of PheCodes of physical conditions to determine effects of suicide death vs. control status (suicide death effect, independent of effects of presence of nonfatal suicidality)) and also on presence/absence of suicidality (presence of SI/SB effect, independent of suicide mortality status). **The table shows that, contrary to neuropsychiatric conditions, physical medical conditions, with some notable exceptions, are more strongly associated with actual suicide mortality than with presence of nonfatal suicidality.**

PheCode	Grouping	Effect: suicide death (p-value)	Effect: presence SI/SB (p-value)
Tachycardia NOS	Circulatory	**1.32 (1.11E-05)**	1.23 (2.20E-04)
Acute pancreatitis	Digestive	**1.75 (2.35E-08)**	1.15 (0.161)
Peptic ulcer	Digestive	**1.68 (6.72E-10)**	1.29 (0.0022)
Hematemesis	Digestive	**1.63 (4.29E-05)**	1.42 (0.0027)
Esophagitis, GERD, and related diseases	Digestive	**1.35 (7.36E-05)**	0.84 (0.0087)
Diseases of teeth and supporting structures	Digestive	1.07 (0.437)	**1.57 (2.15E-08)**
Acidosis	Endocrine	**1.83 (8.59E-16)**	**1.44 (4.69E-07)**
Hypopotassemia	Endocrine	**1.77 (2.48E-20)**	**1.42 (2.41E-09)**
Protein-calorie malnutrition	Endocrine	**1.53 (7.01E-07)**	1.25 (0.0070)
Hypovolemia	Endocrine	**1.48 (3.23E-14)**	1.05 (0.257)
Acute renal failure	Genitourinary	**1.53 (1.25E-08)**	1.09 (0.257)
Renal dialysis	Genitourinary	1.39 (0.0033)	**1.81 (2.52E-09)**
Urinary tract infection	Genitourinary	**1.27 (2.39E-05)**	0.85 (0.0015)
Viral hepatitis C	Infectious	1.36 (0.0047)	**2.60 (2.33E-19)**
Crushing or internal injury to organs	Injuries	**1.84 (4.19E-11)**	0.91 (0.238)
Adverse drug events or drug allergies	Injuries	**1.78 (2.66E-20)**	**1.98 (1.37E-31)**
Fracture of ribs	Injuries	**1.62 (3.01E-08)**	1.09 (0.291)
Fracture of vertebral column w/o spinal cord injury	Injuries	**1.59 (5.14E-06)**	0.91 (0.277)
Open wounds of extremities	Injuries	**1.58 (1.39E-17)**	**1.46 (3.19E-14)**
Concussion	Injuries	**1.55 (1.99E-10)**	0.99 (0.807)
Complication of internal orthopedic device	Injuries	**1.44 (2.62E-05)**	**0.68 (2.99E-06)**
Sprains and strains of back and neck	Injuries	**1.43 (1.72E-14)**	1.15 (0.00104)
Open wound of hand	Injuries	**1.40 (2.32E-06)**	1.05 (0.498)
Burns	Injuries	**1.39 (4.76E-05)**	0.96 (0.596)
Superficial injury w/o infection	Injuries	**1.39 (1.36E-11)**	**1.20 (3.86E-05)**
Contusion	Injuries	**1.37 (1.88E-13)**	1.08 (0.0456)
Open wound of head and face	Injuries	**1.32 (1.27E-07)**	1.06 (0.211)
Skull and face fracture and other intercranial injury	Injuries	**1.31 (2.22E-05)**	**1.33 (9.39E-05)**
Personal history of medication allergy	Injuries	1.25 (0.0184)	**1.94 (1.18E-13)**
Injury, NOS	Injuries	**1.23 (2.36E-06)**	1.04 (0.366)
Other and unspecified disc disorder	Musculoskeletal	**1.60 (3.88E-08)**	0.77 (0.0015)
Degeneration of intervertebral disc	Musculoskeletal	**1.32 (6.00E-07)**	**0.79 (1.15E-05)**
Spondylosis w/o myelopathy	Musculoskeletal	**1.29 (2.70E-05)**	**0.76 (7.89E-07)**
Displacement of intervertebral disc	Musculoskeletal	**1.28 (4.76E-05)**	0.81 (2.63E-04)
Chronic pain syndrome	Neurologic	**2.13 (5.09E-12)**	**1.66 (6.23E-06)**
Convulsions	Neurologic	**1.71 (1.86E-16)**	**1.36 (6.82E-07)**
Encephalopathy	Neurologic	**1.59 (1.16E-06)**	**1.67 (2.59E-08)**
Unspecified disorders of nervous system	Neurologic	**1.58 (9.20E-06)**	**1.53 (2.71E-05)**
Coma	Neurologic	**1.56 (8.14e-05)**	**2.18 (5.78E-12)**

© 2025 Coon H et al. *JAMA Network Open*.

Insomnia	Neurologic	**1.44 (1.64E-12)**	**2.53 (1.92E-88)**
Chronic pain conditions	Neurologic	**1.43 (3.27E-10)**	**1.50 (2.26E-15)**
Abnormal involuntary movements	Neurologic	**1.40 (1.44E-05)**	**1.44 (8.44E-07)**
Migraine	Neurologic	**1.35 (1.49E-06)**	1.21 (8.22E-04)
Other sleep disorders	Neurologic	0.84 (0.0363)	**1.42 (1.52E-06)**
Pneumonitis due to inhalation of food or vomit	Respiratory	**2.63 (5.14E-25)**	**2.18 (2.10E-15)**
Respiratory failure	Respiratory	**2.09 (3.50E-23)**	1.28 (6.11E-04)
Chronic airway obstruction	Respiratory	**1.62 (9.79E-08)**	1.11 (0.259)
Asphyxia and hypoxemia	Respiratory	**1.32 (4.77E-05)**	0.99 (0.767)
Rhabdomyolysis	Symptoms[1]	**2.50 (3.10E-13)**	**2.82 (3.51E-14)**
Syncope and collapse	Symptoms	**1.28 (2.21E-05)**	1.10 (0.0699)
Cervicalgia	Symptoms	**1.28 (3.15E-06)**	0.96 (0.322)

1 Symptoms is a PheWas category including symptoms resulting from conditions across multiple clinical domains

Notes. Odds ratios were derived from logistic regressions.[37] Each binary presence/absence PheCode was regressed on case-control status (suicide death effect) and then on presence/absence of SI/SB (presence SI/SB effect), adjusting for age, sex, and transformed overall number of PheCodes. Tests that were significant after adjustment for multiple testing are shown in bold type.

© 2025 Coon H et al. *JAMA Network Open*.

eTable 3. Clinical Phecode results from previous published studies of Utah suicide mortality cohorts with (SD-S) and without (SD-N) prior nonfatal suicidality with protective effects (SI/SB; Coon et al., 2025)[15]

where logistic regression results showed negative (protective) effects of the presence of nonfatal suicidality or suicide death. **These results appear to reflect conditions that may be associated with care-seeking rather than fatal or nonfatal suicidality.**

**PheCode**	**Grouping**	**Odds ratio: suicide death**	**p-value**	**Odds ratio: presence SI/SB**	**p-value**
Atrial fibrillation	Circulatory	1.17	1.04E-01	**0.64**	**4.52E-06**
Encounter for long-term (current) use of anticoagulants	Circulatory	1.38	1.12E-01	**0.59**	**1.35E-11**
Coronary atherosclerosis	Circulatory	1.09	2.81E-01	**0.63**	**9.87E-09**
Other venous embolism and thrombosis	Circulatory	1.08	3.67E-01	**0.72**	**3.73E-05**
Hemorrhoids	Circulatory	0.93	1.65E-01	**0.80**	**1.31E-05**
Encounter for long-term (current) use of aspirin	Circulatory	0.84	3.85E-02	**0.70**	**4.45E-06**
Cardiomegaly	Circulatory	0.78	8.66E-03	**0.59**	**4.85E-10**
Rash and other nonspecific skin eruption	Dermatologic	**0.75**	**8.35E-06**	0.84	1.08E-03
Atopic/contact dermatitis due to other or unspecified	Dermatologic	**0.72**	**4.03E-08**	**0.75**	**4.58E-08**
Actinic keratosis	Dermatologic	**0.68**	**6.07E-06**	**0.49**	**2.17E-18**
Acne	Dermatologic	**0.64**	**1.83E-08**	0.95	4.65E-01
Sebaceous cyst	Dermatologic	**0.62**	**1.33E-06**	**0.66**	**5.36E-07**
Seborrheic keratosis	Dermatologic	**0.54**	**7.13E-13**	**0.48**	**3.49E-22**
Other dyschromia	Dermatologic	**0.40**	**1.13E-16**	**0.58**	**5.58E-10**
Other diseases of the teeth and supporting structures	Digestive	1.071	4.37E-01	**1.57**	**2.15E-08**
Diverticulosis	Digestive	0.91	1.81E-01	**0.72**	**9.66E-07**
Dysphagia	Digestive	0.91	1.81E-01	**0.75**	**6.34E-06**
Bariatric surgery	Digestive	**0.66**	**1.76E-09**	**0.73**	**2.29E-07**
Hyperlipidemia	Endocrine	**0.78**	**1.76E-05**	**0.75**	**3.32E-08**
Morbid obesity	Endocrine	**0.73**	**1.29E-05**	0.93	2.38E-01
Overweight, obesity and other hyperalimentation	Endocrine	**0.70**	**6.72E-06**	1.23	2.11E-03
Obesity	Endocrine	**0.64**	**1.78E-14**	0.97	5.10E-01
Other disorders of the kidney and ureters	Genitourinary	1.06	4.85E-01	**0.74**	**3.84E-05**
Other anemias	Hematopoietic	1.15	2.03E-02	**0.68**	**5.30E-12**
Thrombocytopenia	Hematopoietic	1.09	3.72E-01	**0.69**	**3.77E-05**
Lymphadenitis	Hematopoietic	0.80	2.33E-03	**0.72**	**1.96E-07**
Septicemia	Infectious	1.12	2.15E-01	**0.64**	**2.26E-07**
Fever of unknown origin	Infectious	0.89	2.53E-02	**0.63**	**2.16E-22**
Bacterial infection NOS	Infectious	0.85	1.18E-01	**0.64**	**5.46E-07**
Conjunctivitis, infectious	Infectious	**0.65**	**7.69E-09**	**0.76**	**1.16E-05**
Influenza	Infectious	**0.64**	**4.90E-07**	0.77	2.74E-04
Viral warts & HPV	Infectious	**0.64**	**6.78E-08**	**0.64**	**1.03E-10**
Sprains and strains	Injuries	1.01	7.63E-01	**0.84**	**1.25E-05**
Dislocation	Injuries	0.95	3.87E-01	**0.67**	**1.01E-12**
Internal derangement of knee	Injuries	0.87	5.70E-02	**0.66**	**1.91E-10**
Joint/ligament sprain	Injuries	0.86	3.29E-02	**0.77**	**2.12E-05**
Fracture of unspecified bones	Injuries	0.85	1.36E-02	**0.63**	**6.79E-16**

© 2025 Coon H et al. *JAMA Network Open*.

Other derangement of joint	Injuries	0.84	2.86E-02	**0.66**	**4.01E-09**
Allergies, other	Injuries	**0.69**	**3.40E-05**	1.12	1.46E-01
Arthropathy NOS	Musculoskeletal	1.09	2.14E-01	**0.76**	**5.75E-05**
Osteopenia or other disorder of bone and cartilage	Musculoskeletal	1.04	5.85E-01	**0.63**	**1.60E-10**
Osteoarthrosis NOS	Musculoskeletal	1.00	9.51E-01	**0.77**	**6.66E-06**
Osteoarthritis; localized	Musculoskeletal	0.99	9.20E-01	**0.65**	**2.18E-09**
Joint effusions	Musculoskeletal	0.97	6.19E-01	**0.63**	**1.71E-13**
Peripheral enthesopathies and allied syndromes	Musculoskeletal	0.90	9.71E-02	**0.72**	**1.26E-07**
Symptoms and disorders of the joints	Musculoskeletal	0.89	1.91E-01	**0.57**	**2.02E-12**
Synovitis and tenosynovitis	Musculoskeletal	0.86	6.51E-02	**0.68**	**8.30E-08**
Pain in joint	Musculoskeletal	**0.83**	**1.38E-05**	**0.70**	**1.99E-19**
Pain in limb	Musculoskeletal	**0.81**	**2.97E-06**	**0.82**	**1.52E-06**
Other disorders of bone and cartilage	Musculoskeletal	0.76	1.67E-03	**0.64**	**1.96E-08**
Osteoarthrosis, localized, primary	Musculoskeletal	**0.71**	**1.12E-05**	**0.69**	**8.43E-08**
Enthesopathy	Musculoskeletal	**0.64**	**1.71E-08**	**0.60**	**8.97E-14**
Benign neoplasm of colon	Neoplasms	**0.73**	**2.45E-07**	**0.70**	**1.77E-09**
Neoplasm of uncertain behavior of skin	Neoplasms	**0.69**	**8.17E-05**	**0.58**	**6.58E-11**
Acquired absence of breast	Neoplasms	**0.67**	**1.88E-05**	0.82	1.21E-02
Neoplasm of uncertain behavior	Neoplasms	**0.60**	**1.28E-09**	**0.56**	**1.96E-15**
Benign neoplasm of skin	Neoplasms	**0.55**	**1.15E-22**	**0.54**	**6.53E-32**
Radiotherapy	Neoplasms	**0.51**	**2.32E-13**	**0.67**	**9.06E-08**
Screening for malignant neoplasms of the skin	Neoplasms	**0.41**	**3.86E-43**	**0.79**	**1.45E-05**
Chemotherapy	Neoplasms	**0.40**	**3.28E-28**	**0.60**	**2.32E-14**
Sleep disorders	Neurologic	0.84	3.63E-02	**1.42**	**1.52E-06**
Obstructive sleep apnea	Neurologic	**0.75**	**1.74E-05**	0.93	2.15E-01
Obstetrical/birth trauma	Pregnancy conditions	**0.61**	**2.12E-07**	**0.61**	**7.97E-10**
Other conditions or status of the mother complicating pregnancy, childbirth, or the puerperium	Pregnancy conditions	**0.55**	**3.08E-09**	**0.59**	**4.04E-10**
Other complications of pregnancy NEC	Pregnancy conditions	**0.50**	**4.38E-08**	**0.61**	**1.72E-06**
Acute bronchitis and bronchiolitis	Respiratory	0.86	6.82E-03	**0.82**	**4.52E-05**
Other upper respiratory disease	Respiratory	0.83	2.40E-03	**0.77**	**9.60E-07**
Acute sinusitis	Respiratory	**0.74**	**1.80E-08**	**0.70**	**3.21E-14**
Allergic rhinitis	Respiratory	**0.70**	**3.09E-08**	**0.73**	**1.96E-08**
Acute upper respiratory infections of multiple or unspecified sites	Respiratory	**0.67**	**3.42E-17**	**0.75**	**1.72E-12**
Cough	Respiratory	**0.64**	**1.31E-19**	**0.79**	**2.06E-08**
Acute pharyngitis	Respiratory	**0.64**	**7.17E-21**	**0.82**	**1.01E-06**
Suppurative and unspecified otitis media	Sense organs	0.80	5.00E-04	**0.69**	**2.34E-11**
Presbyopia	Sense organs	**0.66**	**1.92E-06**	0.88	1.11E-01
Astigmatism	Sense organs	**0.55**	**1.46E-15**	0.84	5.01E-03
Myopia	Sense organs	**0.49**	**1.30E-23**	**0.76**	**3.47E-06**
Thoracic or lumbosacral neuritis or radiculitis, unspecified	Symptoms	1.20	1.04E-02	**0.76**	**3.89E-05**
Musculoskeletal symptoms referable to limbs	Symptoms	1.04	6.63E-01	**0.66**	**4.68E-07**
Abdominal pain	Symptoms	1.03	5.53E-01	**0.84**	**9.83E-06**
Malaise and fatigue	Symptoms	0.99	8.46E-01	**0.84**	**2.91E-05**

© 2025 Coon H et al. *JAMA Network Open*.

Edema	Symptoms	0.97	6.74E-01	**0.75**	**2.06E-06**
Muscle weakness	Symptoms	0.91	3.60E-01	**0.62**	**6.92E-07**

Notes. Odds ratios were derived from logistic regressions.[37] Each binary presence/absence PheCode was regressed on case-control status (suicide death effect) and then on presence/absence of SI/SB (presence SI/SB effect), adjusting for age, sex, and transformed overall number of PheCodes. Tests that were significant after adjustment for multiple testing are shown in bold type.

© 2025 Coon H et al. *JAMA Network Open*.

eTable 4. Comparisons of basic demographics between suicide deaths with vs. without available clinical data and genotyping

**Available data in USMRS suicides**	**N**	**% male**	**Mean age at death (std. dev.)**
Full cohort (1998-2022)	15,742	77.8%	41.09 (17.66)
With clinical data (diagnostic codes, NLP)	5,043	71.8%	42.98 (16.99)
With genotyping	3,401	73.9%	42.27 (17.38)
Cohort of primary focus in the study (>80% European, no related individuals)	2,769	75.1%	43.31 (17.52)

© 2025 Coon H et al. *JAMA Network Open*.

eTables 5-10. Information for NLP manual validation at the individual note level. The UUHSC “cases” in this validation comprised 22 suicide deaths (11 male, 11 female) with evidence from ICD-9 or ICD-10 codes for at least one previous instance of non-lethal suicidal behaviors, including attempts and/or ideation prior to the fatal attempt. These cases were hypothesized to have positive mention of suicidality from the NLP.

The “controls” were selected from a larger pool of population controls in our ongoing studies where there were at least two encounters resulting in an ICD-9 or ICD-10 code of depression, but no ICD-9 or ICD-10 codes indicating suicide attempt or suicidal ideation. *Controls were matched for sex, and were within 2 years to suicide deaths; average age at death for suicides was 30.0 years (standard deviation=9.7 years).* Because of multiple depression diagnoses, controls enriched to potentially have relevant notes for the NLP, but had unknown positivity of suicidality mentions in the notes. All cases and controls were White of non-Hispanic ethnicity.

eTable 5. Characteristics of EHR notes in the manual validation of the NLP

Notes were labeled according to source (psychiatric note vs. not psychiatric note) so that results could be characterized according to psychiatric vs. more general note source. Numbers and sources of notes are described in supplemental table S3.

We used the original lexicon developed for this NLP, with the exception that we removed “sa” and “si” occurring without other text. These terms created false positives in notes from non-psychiatric encounters.

Note Characteristic	Cases	Controls	Total
Total N of notes	6,249	9,099	15,349
N of psychiatric encounter notes	4,750 (76%)	6,096 (67%)	10,846
N of notes from other sources	1,500 (24%)	3,003 (33%)	4,503
N notes manually annotated	293	111	404
N psych notes manually annotated	222	53	275
N other notes manually annotated	71	58	129

© 2025 Coon H et al. *JAMA Network Open*.

eTable 6. Validation details of the NLP within individual notes by note type (psychiatric vs. non-psychiatric encounter)
As expected, notes for suicides with ICD diagnoses of prior non-fatal suicidality (cases) showed more true positive instances of suicidality from the NLP (60.75% for Si and 40.27% for SB) than the matched controls with depression but no ICD diagnoses of suicidality (24.32% for SI and 17.12% for SB). Notes also appeared to be more clearly either positive or negative among cases, with low instances of false positives and no instances of false negatives. Among controls, false positive and negative instances were observed, and ranged from 1% to 8%. Across both cases and controls, false positive rates were approximately 3% and false negative rates were approximately 1%.

Validation Attribute	Cases	Controls	Total
True Positive SI, psych notes	139/222 = 62.61%	18/53 = 33.96%	157/275 = 57.09%
False Positive SI, psych notes	1/222 = 0.45%	0/53 = 0 %	1/275 = 0.36%
True Positive SI, non-psych notes	39/71 = 54.92%	9/58 = 15.12%	48/129 = 37.21%
False Positive SI, non-psych notes	4/71 = 5.63%	6/58 = 10.34%	10/129 = 7.75%
True Positive SI, all notes	178/293 = 60.75%	27/111 = 24.32%	205/404 = 50.74%
False Positive SI, all notes	5/293 = 1.71%	6/111 = 5.41%	11/404 = 2.72%
True Positive SB, psych notes	89/222 = 40.10%	15/53 = 28,30%	104/275 = 37.82%
False Positive SB, psych notes	2/222 = 0.90%	6/53 = 11.32%	8/275 = 2.91%
True Positive SB, non-psych notes	29/71 = 40.84%	4/58 = 6.90%	33/129 = 25.58%
False Positive SB, non-psych notes	2/71 = 2.82%	3/58 = 5.17%	5/129 = 3.88%
True Positive SB, all notes	118/293 = 40.27%	19/111 = 17.12%	137/404 = 33.91%
False Positive SB, all notes	4/293 = 0.68%	9/111 = 8.12%	13/404 = 3.22%
True Negative SI, psych notes	80/222 = 36.04%	34/53 = 64.15%	114/275 = 41.45%
False Negative SI, psych notes	0/222 = 0%	3/53 = 5.66%	3/275 = 1.09%
True Negative SI, non-psych notes	30/71 = 42.25%	40/58 = 68.97%	70/129 = 54.26%
False Negative SI, non-psych notes	0/71 = 0%	3/58 = 5.17%	3/129 = 2.33%
True Negative SI, all notes	110/293 = 37.54%	74/111 = 66.67%	184/404 = 45.55%
False Negative SI, all notes	0/293 = 0%	6/111 = 5.41%	6/404 = 1.49%
True Negative SB, psych notes	129/222 = 58.11%	34/53 = 64.15%	163/275 = 59.27%
False Negative SB, psych notes	0/222 = 0%	0/53 = 0%	0/275 = 0%
True Negative SB, non-psych notes	44/71 = 61.97%	45/58 = 77.59%	89/129 = 68.99%
False Negative SB, non-psych notes	0/71 = 0%	1/58 = 1.72%	1/129 = 0.78%
True Negative SB, all notes	173/293 = 59.04%	79/111 = 71.17%	252/404 = 62.38%
False Negative SB, all notes	0/293 = 0%	1/111 = 0.90%	1/404 = 0.25%

© 2025 Coon H et al. *JAMA Network Open*.

eTable 7. Rater agreement details and kappa statistics

Rater agreement was generally high, and did not differ substantially by note type (psychiatric vs. non-psychiatric).

**Rater attribute**	**Cases**	**Controls**	**Total**
Rater agreement SI, psych notes	178/222 = 80.18%	50/53 = 94.34%	228/275 = 82.91%
Rater agreement SI, non-psych notes	57/71 = 80.28%	54/58 = 93.10%	111/129 = 86.05%
Rater agreement SI, all notes	235/293 = 80.20%	104/111 = 93.60%	339/404 = 83.91%
Rater agreement SB, psych notes	192/222 = 86.49%	48/53 = 90.57%	240/275 = 87.27%
Rater agreement SB, non-psych notes	53/71 = 74.65%	55/58 = 94.83%	108/129 = 83.72%
Rater agreement SB, all notes	245/293 = 83.62%	103/111 = 92.79%	348/404 = 86.14%

eTable 8. Kappa statistics for rater agreement

**Note type**	**Cases**	**Controls**	**Total**
SI, psych notes	0.603	0.872	0.678
SI, non-psych notes	0.606	0.862	0.721
SI, all notes	0.604	0.887	0.745
SB, psych notes	0.730	0.811	0.745
SB, non-psych notes	0.493	0.897	0.674
SB, all notes	0.672	0.856	0.723

eTable 9. Final metrics from manual validation of the NLP in individual notes

	SI			SB		
Variable	Cases	Controls	All	Cases	Controls	All
All Note Sources						
Precision[1]	0.973	0.818	0.949	0.967	0.679	0.949
Recall [2]	1.0	0.818	0.972	1.0	0.950	0.993
F1 Score[3]	0.986	0.818	0.960	0.983	0.792	0.971
Psychiatric Notes						
Precision[1]	0.993	1.0	0.994	0.978	0.714	0.929
Recall [2]	1.0	0.857	0.963	1.0	0.833	0.946
F1 Score[3]	0.996	0.923	0.978	0.989	0.769	0.937
Other Non-Psych Notes						
Precision[1]	0.907	0.600	0.828	0.936	0.571	0.868
Recall [2]	1.0	0.750	0.972	1.0	0.571	0.917
F1 Score[3]	0.951	0.667	0.894	0.967	0.571	0.892

Note. Precision= positive predictive value = (true positive)/(true positive + false positive). Recall = sensitivity = true positive/(true positive + false negative). F1 score = 2*(precision*recall)/(precision+recall).

Supplemental information for NLP validation aggregating all text for each person rather than considering individual notes. Because our objective for the NLP is to determine suicidality at the person level we revisited our manual validation and determined rates of false positives and negatives and rater agreement when considering the entire corpus of notes for each person as the source text.

© 2025 Coon H et al. *JAMA Network Open*.

eTable 10. NLP performance considering the entire corpus of notes for each person as the source text, rather than individual notes

	SI			SB		
**Variable**	**Cases**	**Controls**	**All**	**Cases**	**Controls**	**All**
True positives	20/22 = 90.91%	7/22=31.82%	27/44=61.36%	21/22=95.46%	7/22=31.82%	28/44=63.64%
False positives	0	1/22=4.55%	1/44=2.27%	0	2/22=9.09%	2/44=4.55%
True negatives	2/22 = 9.09%	14/22=63.64%	16/22=36.36%	1/22=4.55%	13/22=59.09%	14/44=31.82%
Precision	1.0	0.875	0.964	1.0	0.778	0.933
Recall	1.0	1.0	1.0	1.0	1.0	1.0
F1 Score	1.0	0.933	0.982	1.0	0.875	0.966

Note. There were no false negatives at the aggregated person level for either cases or controls, so this attribute is not included in the table. Precision= positive predictive value = (true positive)/(true positive + false positive). Recall = sensitivity = true positive/(true positive + false negative). F1 score = 2*(precision*recall)/(precision+recall).

Using aggregated note text, 21 of the 22 suicide deaths were true positive for SB. The one case that was not positive for SB was positive for SI. All of the suicide deaths that were identified by the NLP as positive for SB were also identified as positive for SI except two cases which were only positive for SB. Therefore, all 22 suicide deaths were positive when considering either SI or SB.

In the control records, though none had diagnostic billing codes for SB or SI, nine were found to be positive by the NLP for SB. Eight controls were also positive for SI. Manual review indicated two of the positive controls for SB were false positives, and one of the positive controls for SI was a false positive. These false positives occurred in notes where suicide terms in the lexicon rare or complex, or were negated in complex or non-standard phrasing.

Of note, defining suicidality as either SB or SI, the true positive rate was 29/44 = 65.91%, the false positive rate was 3/44 = 6.82%, and the false negative rate was 0. Precision for this overall definition was therefore 0.936 and the F1 score was 0.967.

This validated NLP was applied to all clinical notes to create the SD-N and SD-S subgroups used in this project; NLP results augmented information regarding presence of suicidal thoughts and behaviors in the diagnostic codes. This process is described in more detail in Coon et al., 2025.[15] Regarding diagnostic codes defining these groups, diagnoses that occurred within one week prior to death were excluded to eliminate diagnoses that could be a consequence of the final fatal suicide event to avoid misinterpreting the code as being tied to that final event. This conservative approach may miss some diagnoses with true associations to risk with very proximal timing. We also noted that even with the NLP, it is likely that some individuals in SD-N may have undetected suicidal thoughts or behaviors which are not currently possible to detect.

© 2025 Coon H et al. *JAMA Network Open*.

eAppendix. Additional details of imputation methods

The SNPs assayed in all cohorts (SD-S, SD-N, UK10K controls, and GenScotland controls) were combined. The hg19 genome build was used for all data. SNPs were then retained only if they were genotyped in all of the datasets. The data were assessed using the McCarthy Group Tools (https://www.chg.ox.ac.uk/~wrayner/tools/index.html), and SNPs were deleted if they did not match locations or allele frequencies in the Haplotype Reference Consortium (HRC) reference panel (https://www.sanger.ac.uk/collaboration/haplotype-reference-consortium/). Next, the control datasets were merged and a diagnostic GWAS of UK10K vs. GenScotland was performed. Any SNPs significantly different across the two control datasets were excluded. Two additional SNPs with extreme suicide-control differences (p-value < 1E-50) were also excluded. The combined dataset was then imputed using the HRC reference panel. Following imputation, SNPs with minor allele frequencies <0.01 or with imputation $R^2 < 0.5$ were also excluded.

© 2025 Coon H et al. *JAMA Network Open*.

eTable 11. **Source studies for polygenic score statistics used to test for underlying neuropsychiatric genetic vulnerabilities**

Phenotype (source study)	N source study participants	Notes	Web addresses for summary statistics
Bipolar Disorder[1]	41.917 BD; 371,549 controls	Lifetime BD (ICD-9/ICD-10 diagnoses; EUR)	https://figshare.com/articles/dataset/PGC3_bipolar_disorder_GWAS_summary_statistics/14102594
Major Depressive Disorder[2]	30,618 MDD; 38,200 controls	Lifetime MDD (ICD-10 diagnoses; EUR)	https://ipsych.dk/en/reerach/downloads
Depressed Affect[3]	357,957 depression spectrum symptoms	UKBiobank, Genetics of Personality Consortium (questionnaire, EUR)	https://ctg.cncr.nl/software/summary_statistics
Neuroticism[3]	390,278 (quantitative neuroticism symptoms)	UKBiobank, Genetics of Personality Consortium (questionnaire, EUR)	https://ctg.cncr.nl/software/summary_statistics
Anxiety[4]	12,655 anxiety disorders; 19,225 controls	iPsych, Comprehensive anxiety diagnoses from ICD-10 (GAD, agoraphobia, panic, phobias, mixed anxiety disorder; EUR)	https://ipsych.dk/en/research/downloads/
PTSD[5]	32,428 PTSD; 174,227 controls	EUR subset of meta-analysis of European research resources, non-military ascertainment (multiple diagnostic methods; EUR)	https://figshare.com/articles/dataset/ptsd2019/14672133
Schizophrenia[6]	67,390 SZ; 94,015 controls	SZ, Psychiatric Genetics Consortium and other worldwide data (multiple diagnostic methods; EUR)	https://figshare.com/articles/dataset/scz2022/19426775
Autism[7]	18,381 autism; 27,969 controls	Lifetime autism spectrum disorder (ICD-10; EUR)	https://ipsych.au.dk/downloads/
ADHD[8]	19,099 ADHD; 34,194 controls	Lifetime ADHD (ICD-10 diagnoses; EUR)	https://ipsych.au.dk/downloads/
Alcohol[9]	414,343 individuals (quantitative data)	Drinks per week (UKBiobank, questionnaire; EUR)	https://www.thessgac.org/data
Smoking[9]	518,633 individuals	Lifetime ever smoked (UKBiobank, questionnaire; EUR)	https://www.thessgac.org/data
Alzheimer's Disease[10]	90,338 AD cases; 1,036,225 controls	AD diagnoses, UKBiobank and 13 other studies (multiple diagnostic methods; EUR)	https://ctg.cncr.nl/software/summary_statistics

Notes. BD = Bipolar Disorder; MDD = Major Depressive Disorder; PTSD = Post-Traumatic Stress Disorder; ADHD = Attention Deficit Hyperactivity Disorder; GAD = Generalized Anxiety Disorder; AD = Alzheimer's Disease. EUR = European genetic ancestry. ICD = International Classification of Diseases.

1. Mullins N, Forstner AJ, O'Connell KS, et al; HUNT All-In Psychiatry. 6 Genome-wide association study of more than 40,000 bipolar disorder 7 cases provides new insights into the underlying biology. *Nat Genet*. 2021;53(6):817-829. Medline:34002096 doi:10.1038/s41588-021-00857-4.
2. Als TD, Kurki MI, Grove J, et al. Depression pathophysiology, risk prediction of recurrence and comorbid psychiatric disorders using genome-wide analyses. *Nat Med*. 2023;29(7):1832-1844. Medline:37464041 doi:10.1038/s41591-023-02352-1
3. Nagel M, Jansen PR, Stringer S, et al; 23andMe Research Team. Meta-analysis of genome-wide association studies for neuroticism in 449,484 individuals identifies novel genetic loci and pathways. *Nat Genet*. 2018;50(7):920-927. Medline:29942085 doi:10.1038/s41588-018-0151-7
4. Meier SM, Trontti K, Purves KL, et al. Genetic variants associated with anxiety and stress-related disorders: a genome-wide association study and mouse-model study. 1 *JAMA Psychiatry*. 2019;76(9):924-932. Medline:31116379 doi:10.1001/jamapsychiatry.2019.1119

© 2025 Coon H et al. *JAMA Network Open*.

5. Nievergelt CM, Maihofer AX, Klengel T, et al. International meta-analysis of PTSD genome-wide association studies identifies sex- and ancestry specific genetic risk loci. *Nat Commun*. 2019;10(1):4558. Medline:31594949 doi:10.1038/s41467-019-12576-w
6. Trubetskoy V, Pardiñas AF, Qi T, et al; Indonesia Schizophrenia Consortium; PsychENCODE; Psychosis Endophenotypes International Consortium; SynGO Consortium; Schizophrenia Working Group of the Psychiatric Genomics Consortium. Mapping genomic loci implicates genes and synaptic biology in schizophrenia. *Nature*. 2022;604(7906):502-508. Medline:35396580 doi:10.1038/s41586-022-04434-5
7. Grove J, Ripke S, Als TD, et al; Autism Spectrum Disorder Working Group of the Psychiatric Genomics Consortium; BUPGEN; Major Depressive Disorder Working Group of the Psychiatric Genomics Consortium; 23andMe Research Team. Identification of common genetic risk variants for autism spectrum disorder. *Nat Genet*. 2019;51(3):431-444. Medline:30804558 doi:10.1038/s41588-019-0344-8
8. Demontis D, Walters 1 RK, Martin J, et al; ADHD Working Group of the Psychiatric Genomics Consortium (PGC); Early Lifecourse & Genetic Epidemiology (EAGLE) Consortium; 23andMe Research Team. Discovery of the first genome-wide significant risk loci for attention deficit/hyperactivity disorder. *Nat Genet*. 2019;51(1):63-75. Medline:30478444 doi:10.1038/s41588-018-0269-7
9. Karlsson Linnér R, Biroli P, Kong E, et al; 23and Me Research Team; eQTLgen Consortium; International Cannabis Consortium; Social Science Genetic Association Consortium. Genome-wide association analyses of risk tolerance and risky behaviors in over 1 million individuals identify hundreds of loci and shared genetic influences. *Nat Genet*. 2019;51(2):245-257. Medline:30643258 doi:10.1038/s41588- 018-0309-3
10. Wightman DP, Jansen IE, Savage JE, et al; 23andMe Research Team. A genome-wide association study with 1,126,563 individuals identifies new risk loci for Alzheimer’s disease. *Nat Genet*. 2021;53(9):1276-1282. Medline:34493870 doi:10.1038/s41588-021-00921-z

© 2025 Coon H et al. *JAMA Network Open*.

eTable 12. Correlations among the PGS scores used in this study

Scores were derived using PRSice with covariates as described for the primary results in Table 1.

PGS correlations using all data	Bipolar disorder	MDD	Depressed Affect	Anxiety	PTSD	Neuroticism	Autism	ADHD	Schizophrenia	Alcohol	Smoking	Alzheimer's disease	Height (neutral control)
Bipolar disorder	1	0.20	0.05	0.11	0.09	0.07	0.07	0.07	0.33	0.02	0.05	0.03	0.00
MDD	0.20	1	0.30	0.18	0.15	0.32	0.10	0.18	0.18	-0.02	0.12	0.03	0.00
Depressed Affect	0.05	0.30	1	0.07	0.10	0.78	0.02	0.08	0.05	-0.02	0.10	0.02	-0.01
Anxiety	0.11	0.18	0.07	1	0.14	0.07	0.17	0.21	0.12	-0.01	0.04	0.02	0.01
PTSD	0.09	0.15	0.10	0.14	1	0.09	0.06	0.07	0.12	0.00	0.06	0.02	0.00
Neuroticism	0.07	0.32	0.78	0.07	0.09	1	0.02	0.05	0.08	-0.02	0.07	0.02	-0.01
Autism	0.07	0.10	0.02	0.17	0.06	0.02	1	0.29	0.09	-0.01	0.02	0.01	0.00
ADHD	0.07	0.18	0.08	0.21	0.07	0.05	0.29	1	0.06	0.01	0.10	0.02	0.01
Schizophrenia	0.33	0.18	0.05	0.12	0.12	0.08	0.09	0.06	1	0.02	0.07	0.02	0.00
Alcohol	0.02	-0.02	-0.02	-0.01	0.00	-0.02	-0.01	0.01	0.02	1	0.19	0.00	0.02
Smoking	0.05	0.12	0.10	0.04	0.06	0.07	0.02	0.10	0.07	0.19	1	0.02	0.00
Alzheimer's disease	0.03	0.03	0.02	0.02	0.02	0.02	0.01	0.02	0.02	0.00	0.02	1	-0.01
Height (neutral control)	0.00	0.00	-0.01	0.01	0.00	-0.01	0.00	0.01	0.00	0.02	0.00	-0.01	1

© 2025 Coon H et al. *JAMA Network Open*.

eFigure. Age at death distributions for SD-N and SD-S. Substantial divergence in age at death occurs at age > 50 years. This was chosen as a threshold for subgroup analyses for this moderating variable to maximize sample size in the two subgroups while capturing differences in the distributions.

Both Males and Females

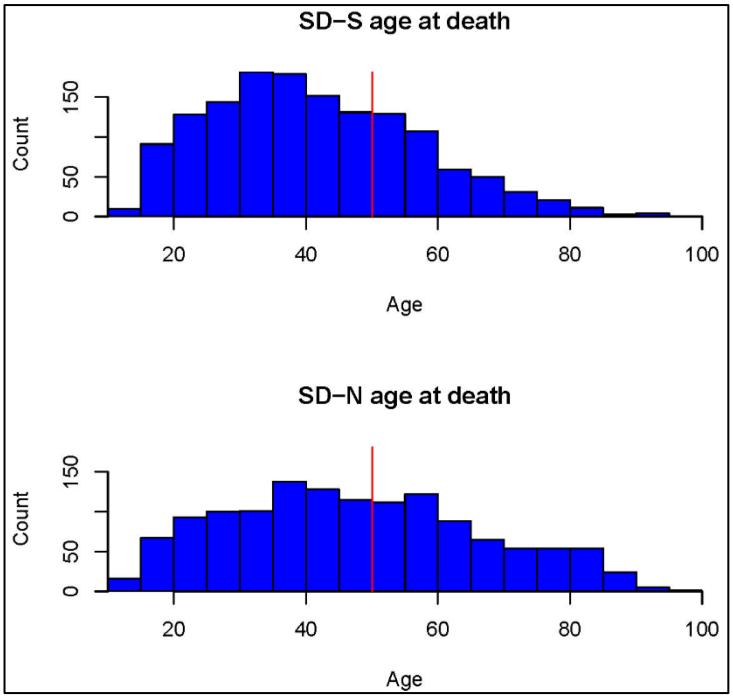

Males only

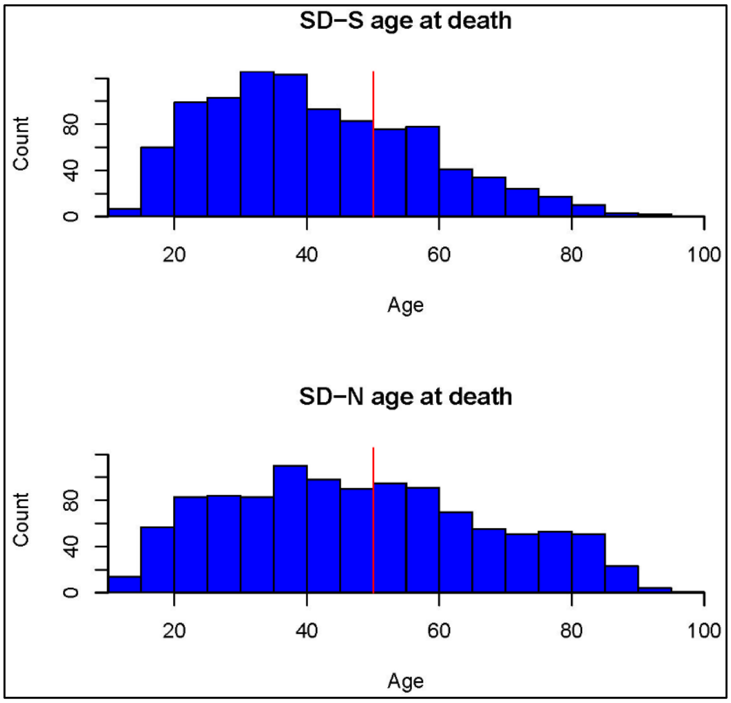

Females only

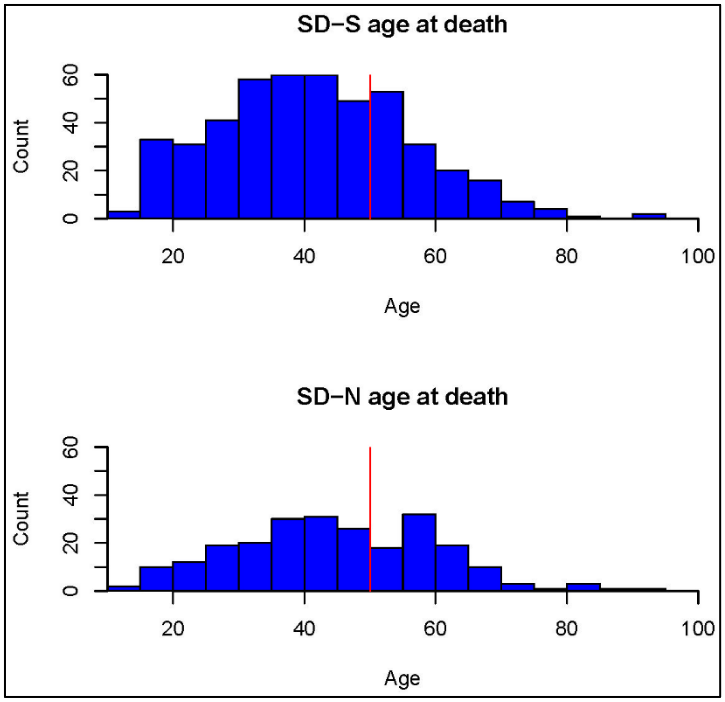

© 2025 Coon H et al. *JAMA Network Open*.

eTable 13. Polygenic score comparisons <u>without</u> adjustment for the moderating variables of sex and age at death

These analyses included suicides with >=80% European ancestry.

Polygenic score for trait or diagnosis	SD-N, mean PGS (SE)	SD-S, mean PGS (SE)	SD-N vs. SD-S, adj. mean difference (95% CI) (95% CI)	p-value	FDR adj. p-value	SD-N vs. Controls, adj. mean difference (95% CI) (95% CI)	p-value	FDR adj. p-value	SD-S vs. controls, adj. mean difference (95% CI) (95% CI)	p-value	FDR adj. p-value
Bipolar Disorder	0.117 (0.022)	0.182 (0.022)	0.063 (0.001-0.125)	**0.045**	0.067	0.289 (0.225-0.353)	**6.26E-19**	**1.88E-18**	0.368 (0.305-0.430)	**1.31E-30**	**3.93E-30**
MDD	0.136 (0.024)	0.246 (0.024)	0.113 (0.047-0.180)	**8.31E-04**	**0.010**	0.317 (0.253-0.381)	**2.61E-22**	**1.04E-21**	0.439 (0.376-0.502)	**1.20E-42**	**7.23E-42**
Depressed Affect	0.036 (0.025)	0.111 (0.024)	0.080 (0.013-0.148)	**0.020**	**0.045**	0.116 (0.052-0.181)	**3.82E-04**	**5.09E-04**	0.186 (0.123-0.249)	**6.89E-09**	**1.38E-08**
Anxiety	0.085 (0.025)	0.184 (0.025)	0.093 (0.023-0.162)	**8.69E-03**	**0.026**	0.253 (0.189-0.317)	**9.88E-15**	**2.37E-14**	0.368 (0.305-0.431)	**2.52E-30**	**6.05E-30**
PTSD	0.130 (0.024)	0.204 (0.024)	0.072 (0.004-0.140)	**0.037**	0.064	0.333 (0.269-0.397)	**1.65E-24**	**9.89E-24**	0.409 (0.346-0.472)	**3.19E-37**	**1.28E-36**
Neuroticism	-0.000 (0.025)	0.103 (0.024)	0.105 (0.037-0.174)	**2.63E-03**	**0.016**	0.057 (-0.007-0.121)	0.083	0.090	0.158 (0.095-0.221)	**9.73E-07**	**1.46E-06**
Autism	0.075 (0.027)	0.088 (0.026)	0.015 (-0.059-0.089)	0.69	0.81	0.156 (0.091-0.221)	**2.18E-06**	**3.74E-06**	0.184 (0.121-0.248)	**1.18E-08**	**2.02E-08**
ADHD	0.089 (0.027)	0.075 (0.026)	-0.008 (-0.082-0.065)	0.83	0.83	0.147 (0.082-0.211)	**8.26E-06**	**1.24E-05**	0.146 (0.082-0.209)	**6.64E-06**	**7.97E-06**
Schizophrenia	0.266 (0.023)	0.344 (0.023)	0.075 (0.010-0.139)	**0.023**	**0.045**	0.672 (0.609-0.735)	**1.73E-96**	**2.08E-95**	0.753 (0.691-0.815)	**4.03E-124**	**4.84E-123**
Alcohol	0.060 (0.026)	0.073 (0.024)	0.012 (-0.058-0.081)	0.74	0.81	0.100 (0.036-0.164)	**2.33E-03**	**2.80E-03**	0.116 (0.053-0.179)	**3.03E-04**	**3.30E-04**
Smoking	0.120 (0.026)	0.072 (0.024)	-0.051 (-0.120-0.018)	0.15	0.20	0.182 (0.118-0.247)	**2.79E-08**	**5.59E-08**	0.148 (0.085-0.211)	**3.96E-06**	**5.29E-06**
Alzheimer's Disease	-0.033 (0.025)	0.063 (0.024)	0.097 (0.029-0.165)	**5.10E-03**	**0.020**	-0.002 (-0.066-0.062)	0.95	0.95	0.094 (0.031-0.157)	**3.43E-03**	**3.43E-03**
**MALES**											
Bipolar Disorder	0.151 (0.025)	0.175 (0.026)	0.028 (-0.044-0.100)	**0.44**	**0.53**	0.318 (0.242-0.395)	4.07E-16	2.44E-15	0.359 (0.279-0.440)	**2.72E-18**	**6.53E-18**
MDD	0.145 (0.027)	0.240 (0.028)	0.102 (0.025-0.179)	**9.44E-03**	**0.053**	0.317 (0.240-0.394)	7.61E-16	3.04E-15	0.428 (0.347-0.509)	**4.08E-25**	**2.45E-24**
Depressed Affect	0.048 (0.027)	0.127 (0.029)	0.083 (0.005-0.161)	**0.038**	**0.092**	0.151 (0.074-0.229)	1.26E-04	1.89E-04	0.222 (0.141-0.304)	**8.16E-08**	**1.63E-07**
Anxiety	0.073 (0.027)	0.179 (0.030)	0.101 (0.021-0.181)	**0.013**	**0.053**	0.264 (0.187-0.341)	1.99E-11	4.77E-11	0.392 (0.311-0.474)	**3.68E-21**	**1.10E-20**
PTSD	0.096 (0.026)	0.201 (0.029)	0.105 (0.028-0.182)	**7.88E-03**	**0.053**	0.308 (0.232-0.385)	3.46E-15	1.04E-14	0.425 (0.344-0.506)	**9.12E-25**	**3.65E-24**
Neuroticism	0.010 (0.028)	0.094 (0.029)	0.086 (0.007-0.164)	**0.032**	**0.092**	0.081 (0.004-0.159)	0.041	0.044	0.164 (0.082-0.245)	**7.94E-05**	**1.06E-04**

© 2025 Coon H et al. *JAMA Network Open*.

Autism	0.041 (0.030)	0.110 (0.031)	0.078 (-0.007-0.163)	**0.07**	**0.12**	0.123 (0.045-0.202)	1.98E-03	2.64E-03	0.220 (0.139-0.302)	**1.30E-07**	**2.23E-07**
ADHD	0.091 (0.030)	0.082 (0.032)	-0.005 (-0.091-0.081)	**0.908**	**0.908**	0.161 (0.083-0.239)	5.32E-05	9.11E-05	0.168 (0.086-0.250)	**6.00E-05**	**9.00E-05**
Schizophrenia	0.247 (0.025)	0.322 (0.027)	0.072 (-0.001-0.145)	**0.053**	**0.106**	0.643 (0.568-0.719)	1.05E-61	1.26E-60	0.712 (0.632-0.792)	**1.45E-67**	**1.75E-66**
Alcohol	0.071 (0.028)	0.092 (0.029)	0.021 (-0.058-0.100)	**0.60**	**0.66**	0.092 (0.014-0.170)	0.020	0.024	0.123 (0.041-0.204)	**3.12E-03**	**3.40E-03**
Smoking	0.126 (0.029)	0.057 (0.030)	-0.071 (-0.153-0.010)	**0.09**	**0.13**	0.184 (0.106-0.262)	3.55E-06	7.09E-06	0.131 (0.050-0.213)	**1.59E-03**	**1.91E-03**
Alzheimer's Disease	-0.013 (0.027)	0.049 (0.029)	0.063 (-0.015-0.141)	**0.12**	**0.15**	0.018 (-0.059-0.095)	0.65	0.65	0.081 (-0.001-0.162)	**0.052**	**0.052**
**FEMALES**											
Bipolar Disorder	0.007 (0.051)	0.214 (0.040)	0.207 (0.076-0.338)	**1.99E-03**	**0.024**	0.007 (-0.121-0.134)	0.92	0.92	0.214 (0.122-0.306)	**5.25E-06**	**2.10E-05**
MDD	0.163 (0.056)	0.279 (0.042)	0.117 (-0.023-0.257)	**0.10**	**0.25**	0.163 (0.034-0.291)	0.013	0.039	0.279 (0.187-0.372)	**2.85E-09**	**1.71E-08**
Depressed Affect	-0.015 (0.058)	0.079 (0.042)	0.094 (-0.047-0.235)	**0.19**	**0.39**	-0.015 (-0.143-0.113)	0.82	0.90	0.079 (-0.013-0.171)	**0.09**	**0.11**
Anxiety	0.132 (0.062)	0.205 (0.042)	0.073 (-0.072-0.218)	**0.32**	**0.49**	0.132 (0.004-0.261)	0.043	0.104	0.205 (0.113-0.297)	**1.27E-05**	**3.80E-05**
PTSD	0.197 (0.058)	0.168 (0.043)	-0.029 (-0.173-0.115)	**0.69**	**0.89**	0.197 (0.069-0.325)	2.55E-03	0.015	0.168 (0.076-0.260)	**3.52E-04**	**8.44E-04**
Neuroticism	-0.057 (0.058)	0.119 (0.044)	0.176 (0.031-0.321)	**0.018**	**0.071**	-0.057 (-0.185-0.071)	0.38	0.46	0.119 (0.027-0.211)	**0.012**	**0.023**
Autism	0.187 (0.059)	0.052 (0.047)	-0.134 (-0.287-0.018)	**0.08**	**0.25**	0.187 (0.059-0.315)	4.27E-03	0.017	0.052 (-0.040-0.145)	**0.27**	**0.27**
ADHD	0.114 (0.062)	0.089 (0.045)	-0.026 (-0.176-0.125)	**0.74**	**0.89**	0.114 (-0.014-0.243)	0.08	0.15	0.089 (-0.004-0.181)	**0.059**	**0.079**
Schizophrenia	0.289 (0.052)	0.371 (0.042)	0.082 (-0.053-0.218)	**0.23**	**0.40**	0.289 (0.161-0.417)	9.79E-06	1.17E-04	0.371 (0.279-0.463)	**2.97E-15**	**3.57E-14**
Alcohol	0.068 (0.065)	0.063 (0.043)	-0.006 (-0.154-0.143)	**0.94**	**0.97**	0.068 (-0.060-0.197)	0.30	0.39	0.063 (-0.029-0.155)	**0.18**	**0.20**
Smoking	0.112 (0.059)	0.110 (0.041)	-0.003 (-0.142-0.137)	**0.97**	**0.97**	0.112 (-0.016-0.240)	0.09	0.15	0.110 (0.018-0.202)	**0.020**	**0.034**
Alzheimer's Disease	-0.084 (0.055)	0.100 (0.041)	0.183 (0.047-0.320)	**8.54E-03**	**0.051**	-0.084 (-0.212-0.044)	0.20	0.30	0.100 (0.008-0.192)	**0.034**	**0.050**
**AGE AT DEATH ≤ 50**											
Bipolar Disorder	0.132 (0.030)	0.188 (0.026)	0.055 (-0.024-0.133)	**0.17**	**0.35**	0.305 (0.225-0.385)	7.19E-14	2.16E-13	0.371 (0.300-0.442)	**2.39E-24**	**7.18E-24**
MDD	0.167 (0.032)	0.283 (0.028)	0.120 (0.036-0.203)	**4.97E-03**	**0.030**	0.339 (0.259-0.419)	1.08E-16	4.34E-16	0.477 (0.405-0.548)	**6.54E-39**	**3.93E-38**
Depressed Affect	0.024 (0.033)	0.125 (0.028)	0.106 (0.020-0.192)	**0.015**	**0.061**	0.101 (0.021-0.181)	0.014	0.018	0.202 (0.130-0.273)	**3.67E-08**	**7.34E-08**

© 2025 Coon H et al. *JAMA Network Open*.

Anxiety	0.124 (0.032)	0.182 (0.030)	0.056 (-0.032-0.144)	**0.21**	**0.36**	0.294 (0.214-0.374)	6.57E-13	1.58E-12	0.369 (0.298-0.441)	**6.18E-24**	**1.48E-23**
PTSD	0.160 (0.031)	0.250 (0.029)	0.094 (0.009-0.179)	**0.030**	**0.090**	0.368 (0.288-0.448)	1.71E-19	1.03E-18	0.459 (0.387-0.530)	**3.50E-36**	**1.40E-35**
Neuroticism	-0.013 (0.033)	0.117 (0.029)	0.138 (0.051-0.224)	**1.79E-03**	**0.021**	0.049 (-0.032-0.129)	0.24	0.24	0.175 (0.103-0.246)	**1.94E-06**	**2.58E-06**
Autism	0.075 (0.035)	0.095 (0.031)	0.031 (-0.061-0.123)	**0.51**	**0.69**	0.160 (0.079-0.240)	1.01E-04	1.52E-04	0.187 (0.115-0.259)	**3.52E-07**	**5.29E-07**
ADHD	0.138 (0.034)	0.127 (0.030)	0.001 (-0.090-0.092)	**0.99**	**0.99**	0.189 (0.108-0.269)	4.28E-06	7.33E-06	0.195 (0.123-0.267)	**1.12E-07**	**1.93E-07**
Schizophrenia	0.329 (0.030)	0.352 (0.028)	0.023 (-0.058-0.104)	**0.58**	**0.70**	0.740 (0.662-0.819)	3.39E-75	4.07E-74	0.767 (0.697-0.837)	**7.84E-100**	**9.41E-99**
Alcohol	0.049 (0.034)	0.082 (0.028)	0.029 (-0.057-0.115)	**0.51**	**0.69**	0.094 (0.013-0.174)	0.023	0.027	0.127 (0.055-0.198)	**5.52E-04**	**6.02E-04**
Smoking	0.156 (0.033)	0.095 (0.028)	-0.065 (-0.151-0.021)	**0.14**	**0.33**	0.212 (0.132-0.292)	2.34E-07	4.68E-07	0.167 (0.095-0.239)	**5.13E-06**	**6.16E-06**
Alzheimer's Disease	0.025 (0.031)	0.045 (0.028)	0.017 (-0.067-0.100)	**0.70**	**0.76**	0.060 (-0.020-0.140)	0.14	0.16	0.071 (-0.001-0.143)	**0.05**	**0.05**
**AGE AT DEATH > 50**											
Bipolar Disorder	0.093 (0.033)	0.181 (0.040)	0.095 (-0.008-0.198)	**0.07**	**0.17**	0.269 (0.181-0.357)	1.87E-09	5.61E-09	0.366 (0.265-0.467)	**1.39E-12**	**4.26E-12**
MDD	0.091 (0.037)	0.177 (0.043)	0.110 (-0.003-0.224)	**0.06**	**0.17**	0.280 (0.192-0.368)	4.24E-10	1.70E-09	0.366 (0.265-0.468)	**1.42E-12**	**4.26E-12**
Depressed Affect	0.051 (0.038)	0.072 (0.043)	0.033 (-0.080-0.147)	**0.57**	**0.74**	0.130 (0.042-0.218)	3.89E-03	5.84E-03	0.148 (0.046-0.249)	**4.45E-03**	**7.63E-03**
Anxiety	0.044 (0.038)	0.207 (0.043)	0.166 (0.050-0.281)	**5.00E-03**	**0.020**	0.218 (0.130-0.306)	1.21E-06	2.90E-06	0.384 (0.283-0.485)	**1.08E-13**	**6.47E-13**
PTSD	0.097 (0.038)	0.094 (0.044)	-0.002 (-0.117-0.114)	**0.98**	**0.98**	0.306 (0.218-0.394)	8.95E-12	5.37E-11	0.309 (0.208-0.411)	**2.09E-09**	**5.02E-09**
Neuroticism	0.017 (0.039)	0.071 (0.043)	0.061 (-0.055-0.177)	**0.30**	**0.60**	0.071 (-0.017-0.160)	0.11	0.11	0.125 (0.023-0.227)	**0.016**	**0.021**
Autism	0.074 (0.043)	0.076 (0.048)	0.004 (-0.125-0.132)	**0.96**	**0.98**	0.160 (0.072-0.249)	3.93E-04	7.86E-04	0.171 (0.069-0.273)	**9.84E-04**	**1.97E-03**
ADHD	0.022 (0.042)	-0.044 (0.048)	-0.058 (-0.186-0.070)	**0.37**	**0.64**	0.085 (-0.003-0.174)	0.06	0.07	0.023 (-0.079-0.124)	**0.66**	**0.66**
Schizophrenia	0.180 (0.035)	0.335 (0.042)	0.168 (0.060-0.275)	**2.28E-03**	**0.014**	0.586 (0.499-0.673)	5.85E-40	7.02E-39	0.744 (0.645-0.844)	**4.23E-48**	**5.07E-47**
Alcohol	0.075 (0.040)	0.051 (0.046)	-0.031 (-0.152-0.090)	**0.61**	**0.74**	0.108 (0.020-0.197)	0.016	0.022	0.088 (-0.014-0.189)	**0.09**	**0.10**
Smoking	0.072 (0.040)	0.013 (0.044)	-0.044 (-0.163-0.074)	**0.46**	**0.69**	0.145 (0.057-0.234)	1.30E-03	2.23E-03	0.093 (-0.009-0.195)	**0.07**	**0.09**
Alzheimer's Disease	-0.103 (0.039)	0.099 (0.043)	0.214 (0.096-0.331)	**3.78E-04**	**0.005**	-0.077 (-0.165-0.012)	0.090	0.098	0.130 (0.028-0.232)	**0.012**	**0.018**

Notes. All tests were adjusted for sex, age at death (for suicide cohorts, including the within-age subgroup tests), and 20 genetic ancestry principal components. Results in bold type are significant. FDR was calculated within each cohort test

© 2025 Coon H et al. *JAMA Network Open*.

(SD-N vs. SD-S; SD-N vs. control; SD-S vs. control). All PGS were normalized to the controls, giving the controls mean PGS of 0 and standard deviation of 1.

SE = standard error of the mean. SD-N = suicide deaths with no evidence of prior nonfatal suicidality. SD-S = suicide deaths with evidence of prior nonfatal suicidality. MDD = major depressive disorder. PTSD = post-traumatic stress disorder. ADHD = attention deficit hyperactivity disorder. FDR-adj = adjusted for False Discovery Rate.

© 2025 Coon H et al. *JAMA Network Open*.

eTable 14. Polygenic score (PGS) analysis of covariance results comparing suicide death cohorts within subsets defined by sex and by age at death

**Polygenic scored= for trait or diagnosis**	**SD-N mean PGS (SE)**	**SD-S mean PGS (SE)**	**SD-N vs. SD-S adj. mean difference (95% CI)**	**p-value**	**FDR adj. p-value**	**SD-N vs. Controls adj. mean difference (95% CI)**	**p-value**	**FDR adj. p-value**	**SD-S vs. Controls adj. mean difference (95% CI)**	**p-value**	**FDR adj. p-value**
					**MALES**						
Bipolar disorder	0.157 (0.025)	0.173 (0.026)	0.017 (-0.054-0.088)	0.64	0.64	0.157 (0.095-0.218)	**5.70E-07**	**2.28E-06**	0.173 (0.108-0.238)	**1.74E-07**	**4.17E-07**
MDD	0.158 (0.027)	0.235 (0.028)	0.077 (0.001-0.154)	**0.048**	0.12	0.158 (0.096-0.220)	**5.31E-07**	**2.28E-06**	0.235 (0.170-0.301)	**1.81E-12**	**1.08E-11**
Depressed Affect	0.049 (0.027)	0.126 (0.029)	0.078 (0.000-0.156)	0.050	0.12	0.049 (-0.013-0.110)	0.12	0.17	0.126 (0.061-0.192)	**1.52E-04**	**3.04E-04**
Anxiety	0.076 (0.027)	0.178 (0.030)	0.102 (0.023-0.182)	**0.012**	0.082	0.076 (0.014-0.138)	**0.016**	**0.027**	0.178 (0.112-0.244)	**1.12E-07**	**2.36E-07**
PTSD	0.102 (0.026)	0.199 (0.029)	0.097 (0.020-0.173)	**0.014**	0.082	0.102 (0.041-0.164)	**1.15E-03**	**2.29E-03**	0.199 (0.133-0.264)	**2.81E-09**	**1.12E-08**
Neuroticism	0.012 (0.028)	0.094 (0.029)	0.082 (0.004-0.159)	0.041	0.12	0.012 (-0.050-0.074)	0.70	0.76	0.094 (0.028-0.159)	**5.02E-03**	**6.69E-03**
Autism	0.044 (0.030)	0.109 (0.031)	0.064 (-0.020-0.149)	0.14	0.21	0.044 (-0.018-0.107)	0.16	0.20	0.109 (0.043-0.174)	**1.23E-03**	**2.11E-03**
ADHD	0.106 (0.030)	0.077 (0.032)	-0.029 (-0.114-0.056)	0.50	0.55	0.106 (0.044-0.169)	**8.32E-04**	**2.00E-03**	0.077 (0.011-0.143)	**0.022**	**0.026**
Schizophrenia	0.259 (0.025)	0.318 (0.027)	0.059 (-0.013-0.132)	0.11	0.19	0.259 (0.197-0.320)	**1.53E-16**	**1.84E-15**	0.318 (0.253-0.383)	**1.39E-21**	**1.67E-20**
Alcohol (drinks/ week)	0.066 (0.028)	0.094 (0.029)	0.028 (-0.050-0.107)	0.48	0.55	0.066 (0.004-0.128)	**0.037**	0.056	0.094 (0.029-0.159)	**4.90E-03**	**6.69E-03**
Smoking (ever smoked)	0.132 (0.029)	0.055 (0.029)	-0.077 (-0.158-0.004)	0.063	0.13	0.132 (0.070-0.194)	**3.10E-05**	**9.30E-05**	0.055 (-0.010-0.121)	0.10	0.11
Alzheimer's disease	-0.008 (0.027)	0.047 (0.029)	0.055 (-0.022-0.133)	0.16	0.22	-0.008 (-0.070-0.053)	0.79	0.79	0.047 (-0.018-0.112)	0.16	0.16
Height (neutral control PGS)	-0.048 (0.028)	-0.009 (0.030)	0.039 (-0.041-0.119)	0.34		-0.048 (-0.110-0.014)	0.13		-0.009 (-0.074-0.057)	0.80	
**Polygenic scored= for trait or diagnosis**	**SD-N mean PGS (SE)**	**SD-S mean PGS (SE)**	**SD-N vs. SD-S adj. mean difference (95% CI)**	**p-value**	**FDR adj.p-value**	**SD-N vs. Controls adj. mean difference (95% CI)**	**p-value**	**FDR adj. p-value**	**SD-S vs. Controls adj. mean difference (95% CI)**	**p-value**	**FDR adj. p-value**
					**FEMALES**						
Bipolar disorder	0.008 (0.051)	0.214 (0.040)	0.204 (0.073-0.335)	**2.37E-03**	**0.028**	0.008 (-0.120-0.136)	0.90	0.90	0.212 (0.120-0.304)	**6.23E-06**	**2.49E-05**
MDD	0.167	0.275	0.108 (-	0.13	0.31	0.167	**0.011**	**0.032**	0.275	**4.86E-**	**2.92E-**

© 2025 Coon H et al. *JAMA Network Open*.

	(0.056)	(0.042)	0.031-0.248)			(0.039-0.295)			(0.183-0.367)	**09**	**08**
Depressed Affect	-0.015 (0.058)	0.079 (0.042)	0.093 (-0.048-0.234)	0.20	0.39	-0.015 (-0.143-0.114)	0.82	0.90	0.079 (-0.013 0.1718)	0.09	0.11
Anxiety	0.133 (0.062)	0.204 (0.042)	0.071 (-0.074-0.215)	0.34	0.51	0.133 (0.005-0.262)	**0.042**	0.100	0.204 (0.112-0.296)	**1.39E-05**	**4.18E-05**
PTSD	0.200 (0.058)	0.166 (0.043)	-0.034 (-0.178-0.110)	0.65	0.77	0.200 (0.072-0.328)	**2.26E-03**	**0.016**	0.166 (0.074-0.258)	**4.23E-04**	**1.02E-03**
Neuroticism	-0.056 (0.058)	0.118 (0.044)	0.174 (0.029-0.319)	**0.019**	0.075	-0.056 (-0.184-0.072)	0.39	0.47	0.119 (0.026-0.210)	**0.012**	**0.024**
Autism	0.188 (0.059)	0.051 (0.047)	-0.137 (-0.289-0.015)	0.08	0.24	0.188 (0.060-0.316)	**4.02E-03**	**0.016**	0.051 (-0.041-0.144)	0.28	0.28
ADHD	0.120 (0.062)	0.084 (0.045)	-0.036 (-0.186-0.115)	0.64	0.77	0.120 (-0.009-0.248)	0.07	0.14	0.084 (-0.009-0.176)	0.08	0.101
Schizophrenia	0.293 (0.052)	0367 (0.042)	0.074 (-0.061-0.210)	0.28	0.48	0.293 (0.165-0.421)	**7.27E-06**	**8.73E-05**	0.367 (0.275-0.459)	**5.86E-15**	**7.04E-14**
Alcohol (drinks/ week)	0.067 (0.065)	0.065 (0.043)	-0.002 (-0.150-0.147)	0.98	0.98	0.067 (-0.062-0.195)	0.31	0.41	0.065 (-0.028-0.157)	0.17	0.19
Smoking (ever smoked)	0.114 (0.059)	0.108 (0.041)	-0.007 (-0.146-0.133)	0.92	0.98	0.114 (-0.014-0.243)	0.08	0.14	0.108 (0.016-0.200)	**0.022**	**0.038**
Alzheimer's disease	-0.082 (0.055)	0.098 (0.041)	0.180 (0.044-0.316)	**0.010**	**0.058**	-0.082 (-0.210-0.046)	0.21	0.31	0.099 (0.006-0.190)	**0.036**	0.055
Height (neutral control PGS)	-0.015 (0.059)	0.052 (0.043)	0.067 (-0.077-0.210)	0.36		-0.015 (-0.143-0.114)	0.82		0.052 (-0.040-0.144)	0.27	

**Polygenic score for trait or diagnosis**	**SD-N mean PGS (SE)**	**SD-S mean PGS (SE)**	**SD-N vs. SD-S adj. mean difference (95% CI)**	**p-value**	**FDR adj.p-value**	**SD-N vs. Controls adj. mean difference (95% CI)**	**p-value**	**FDR adj. p-value**	**SD-S vs. Controls adj. mean difference (95% CI)**	**p-value**	**FDR adj. p-value**
**AGE AT DEATH < 50 YEARS**											
Bipolar Disorder	0.123 (0.030)	0.178 (0.026)	0.058 (-0.021-0.137)	0.15	0.31	0.129 (0.056-0.201)	5.01E-04	1.20E-03	0.183 (0.120-0.246)	1.06E-08	3.17E-08
MDD	0.144 (0.032)	0.259 (0.028)	0.102 (0.018-0.186)	**0.017**	0.088	0.155 (0.082-0.227)	**2.92E-05**	**1.75E-04**	0.266 (0.203-0.329)	**1.08E-16**	**6.48E-16**
Depressed Affect	0.023 (0.033)	0.124 (0.028)	0.101 (0.015-0.187)	**0.022**	0.088	0.024 (-0.048-0.097)	0.51	0.61	0.125 (0.062-0.187)	**1.01E-04**	**2.03E-04**
Anxiety	0.118 (0.032)	0.177 (0.030)	0.047 (-0.041-0.136)	0.29	0.50	0.118 (0.045-0.190)	**1.49E-03**	**2.56E-03**	0.176 (0.113-0.239)	**4.26E-08**	**1.02E-07**
PTSD	0.148 (0.031)	0.237 (0.029)	0.092 (0.007-0.178)	**0.034**	0.103	0.133 (0.060-0.205)	**3.31E-04**	**9.92E-04**	0.226 (0.163-0.289)	**1.79E-12**	**7.17E-12**
Neuroticism	-0.017 (0.033)	0.112 (0.029)	0.121 (0.035-0.208)	**0.006**	0.073	-0.019 (-0.092-0.054)	0.61	0.62	0.112 (0.049-0.175)	**4.84E-04**	**8.30E-04**
Autism	0.069 (0.035)	0.088 (0.031)	0.018 (-0.074-0.111)	0.70	0.76	0.070 (-0.003-0.142)	0.061	0.081	0.087 (0.024-0.150)	**6.84E-03**	**8.15E-03**

© 2025 Coon H et al. *JAMA Network Open*.

ADHD	0.110 (0.034)	0.097 (0.030)	-0.023 (-0.114-0.068)	0.61	0.74	0.118 (0.045-0.191)	**1.45E-03**	**2.56E-03**	0.103 (0.040-0.166)	**1.42E-03**	**2.12E-03**
Schizophrenia	0.307 (0.030)	0.329 (0.028)	0.024 (-0.058-0.106)	0.57	0.74	0.296 (0.224-0.369)	**1.15E-15**	**1.38E-14**	0.322 (0.259-0.385)	**1.00E-23**	**1.20E-22**
Alcohol	0.059 (0.034)	0.093 (0.028)	0.035 (-0.051-0.122)	0.42	0.64	0.071 (-0.002-0.144)	0.055	0.081	0.101 (0.038-0.164)	**1.68E-03**	**2.24E-03**
Smoking	0.145 (0.033)	0.084 (0.028)	-0.067 (-0.153-0.020)	0.13	0.31	0.148 (0.075-0.221)	**6.53E-05**	**2.61E-04**	0.086 (0.023-0.149)	**7.47E-03**	**8.15E-03**
Alzheimer's Disease	0.017 (0.031)	0.036 (0.028)	0.013 (-0.071-0.097)	0.76	0.76	0.018 (-0.054-0.091)	0.62	0.62	0.038 (-0.025-0.101)	0.23	0.23
Height (neutral control PGS)	-0.014 (0.034)	-0.010 (0.029)	0.001 (-0.088-0.090)	0.98		-0.017 (-0.090-0.056)	0.65		-0.012 (-0.075-0.051)	0.71	
**Polygenic score for trait or diagnosis**	**SD-N mean PGS (SE)**	**SD-S mean PGS (SE)**	**SD-N vs. SD-S adj. mean difference (95% CI)**	**p-value**	**FDR adj.p-value**	**SD-N vs. Controls adj. mean difference (95% CI)**	**p-value**	**FDR adj. p-value**	**SD-S vs. Controls adj. mean difference (95% CI)**	**p-value**	**FDR adj. p-value**
**AGE AT DEATH > 50 YEARS**											
Bipolar Disorder	0.116 (0.033)	0.199 (0.040)	0.076 (-0.028-0.179)	0.15	0.35	0.124 (0.041-0.207)	**3.34E-03**	**0.013**	0.204 (0.108-0.301)	**3.41E-05**	**1.02E-04**
MDD	0.148 (0.037)	0.223 (0.043)	0.079 (-0.035-0.194)	0.17	0.35	0.159 (0.076-0.242)	**1.84E-04**	**1.10E-03**	0.229 (0.133-0.326)	**3.38E-06**	**2.03E-05**
Depressed Affect	0.054 (0.038)	0.074 (0.043)	0.046 (-0.069-0.161)	0.44	0.58	0.054 (-0.029-0.138)	0.20	0.22	0.074 (-0.023-0.171)	0.13	0.18
Anxiety	0.057 (0.038)	0.218 (0.043)	0.179 (0.063-0.295)	**2.52E-03**	**0.015**	0.054 (-0.029-0.138)	0.20	0.22	0.216 (0.120-0.313)	**1.17E-05**	**4.70E-05**
PTSD	0.128 (0.038)	0.118 (0.044)	0.000 (-0.117-0.117)	0.99	0.99	0.110 (0.027-0.193)	**0.010**	**0.029**	0.108 (0.011-0.204)	**0.029**	0.058
Neuroticism	0.027 (0.039)	0.079 (0.043)	0.084 (-0.033-0.201)	0.16	0.35	0.023 (-0.060-0.107)	0.58	0.58	0.077 (-0.020-0.174)	0.12	0.18
Autism	0.090 (0.043)	0.089 (0.048)	-0.010 (-0.140-0.120)	0.88	0.96	0.090 (0.007-0.174)	**0.034**	0.058	0.089 (-0.008-0.185)	0.07	0.13
ADHD	0.091 (0.042)	0.012 (0.048)	-0.068 (-0.197-0.061)	0.30	0.45	0.099 (0.016-0.182)	**0.020**	**0.040**	0.016 (-0.080-0.113)	0.74	0.74
Schizophrenia	0.233 (0.035)	0.377 (0.042)	0.135 (0.026-0.244)	**0.015**	0.061	0.221 (0.138-0.304)	**1.90E-07**	**2.28E-06**	0.371 (0.274-0.467)	**5.61E-14**	**6.73E-13**
Alcohol	0.050 (0.040)	0.031 (0.046)	-0.025 (-0.146-0.097)	0.69	0.83	0.063 (-0.020-0.147)	0.14	0.18	0.039 (-0.058-0.136)	0.43	0.51
Smoking	0.099 (0.040)	0.034 (0.044)	-0.068 (-0.189-0.053)	0.27	0.45	0.101 (0.018-0.184)	**0.017**	**0.040**	0.036 (-0.061-0.133)	0.47	0.51
Alzheimer's Disease	-0.082 (0.039)	0.116 (0.043)	0.211 (0.093-0.330)	**5.08E-04**	**6.10E-03**	-0.080 (-0.163-0.003)	0.059	0.089	0.117 (0.021-0.214)	**0.017**	**0.042**

© 2025 Coon H et al. *JAMA Network Open*.

Height (neutral control PGS)	-0.062 (0.037)	0.062 (0.046)	0.110 (-0.006-0.227)	0.063		-0.065 (-0.148-0.018)	0.12		0.060 (-0.036-0.157)	0.22	

Note. All tests were adjusted for sex, age at death (in suicide cohorts), and 20 genetic ancestry principal components. Results in bold type were significant. FDR was calculated within each cohort test (SD-N vs. SD-S; SD-N vs. control; SD-S vs. control). Results for height, which was included as a check for proper ancestry adjustment, were not included in the FDR correction for substantive results.

All PGS were normalized to controls, giving the controls mean PGS of zero, and standard deviation of one.

SE = standard error of the mean. SD-N = suicide deaths with no evidence of prior nonfatal suicidality. SD-S = suicide deaths with evidence of prior nonfatal suicidality. MDD = major depressive disorder. PTSD = post-traumatic stress disorder. ADHD = attention deficit hyperactivity disorder. FDR-adj = adjusted for False Discovery Rate.

For males, incremental variance explained (R^2) by PGS controlling for other effects in the model ranged from 0.001% to 0.94%. For females, incremental variance explained (R^2) by PGS controlling for other effects in the model ranged from 0.0002% to 1.30%. For suicide deaths with age at death < 50, incremental variance explained (R^2) by PGS controlling for other effects in the model ranged from 0.001% to 0.49%. For suicide deaths with age at death > 50, incremental variance explained (R^2) by PGS controlling for other effects in the model ranged from 0.0001% to 1.20%.

© 2025 Coon H et al. *JAMA Network Open*.

eTable 15. Polygenic score comparisons including suicides with ≥90% European ancestry; combined males and females

Polygenic score for trait or diagnosis	SD-N, mean PGS (SE)	SD-S, mean PGS (SE)	SD-N vs. SD-S, adj. mean difference (95% CI) (95% CI)	p-value	FDR adj. p-value	SD-N vs. Controls, adj. mean difference (95% CI) (95% CI)	p-value	FDR adj. p-value	SD-S vs. controls, adj. mean difference (95% CI) (95% CI)	p-value	FDR adj. p-value
Bipolar Disorder	0.115 (0.023)	0.186 (0.023)	0.071 (0.007-0.136)	**0.030**	0.072	0.120 (0.063-0.177)	**3.46E-05**	**1.38E-04**	0.191 (0.136-0.245)	**8.47E-12**	**3.39E-11**
MDD	0.138 (0.025)	0.246 (0.024)	0.098 (0.030-0.167)	**5.08E-03**	**0.030**	0.149 (0.092-0.206)	**3.02E-07**	**1.81E-06**	0.253 (0.198-0.308)	**1.78E-19**	**1.07E-18**
Depressed Affect	0.042 (0.025)	0.109 (0.024)	0.073 (0.003-0.142)	**0.041**	0.072	0.043 (-0.014-0.100)	0.14	0.17	0.109 (0.054-0.164)	**9.38E-05**	**1.88E-04**
Anxiety	0.081 (0.025)	0.192 (0.025)	0.108 (0.036-0.179)	**3.15E-03**	**0.030**	0.080 (0.023-0.137)	**6.30E-03**	**0.011**	0.190 (0.135-0.245)	**1.16E-11**	**3.49E-11**
PTSD	0.129 (0.025)	0.197 (0.025)	0.073 (0.003-0.143)	**0.042**	0.072	0.114 (0.056-0.171)	**9.79E-05**	**2.94E-04**	0.186 (0.131-0.241)	**2.98E-11**	**7.15E-11**
Neuroticism	0.005 (0.026)	0.101 (0.025)	0.096 (0.025-0.168)	**7.88E-03**	**0.032**	0.002 (-0.055-0.060)	0.94	0.94	0.100 (0.045-0.154)	**3.78E-04**	**6.48E-04**
Autism	0.072 (0.028)	0.091 (0.027)	0.018 (-0.059-0.095)	0.65	0.65	0.074 (0.016-0.131)	**0.012**	**0.016**	0.090 (0.035-0.145)	**1.39E-03**	**2.09E-03**
ADHD	0.096 (0.027)	0.069 (0.026)	-0.029 (-0.104-0.047)	0.46	0.55	0.104 (0.047-0.161)	**3.89E-04**	**7.78E-04**	0.074 (0.019-0.129)	**8.19E-03**	**0.010**
Schizophrenia	0.276 (0.024)	0.342 (0.024)	0.065 (-0.002-0.132)	0.06	0.09	0.264 (0.207-0.321)	**1.05E-19**	**1.26E-18**	0.336 (0.281-0.391)	**3.79E-33**	**4.55E-32**
Alcohol	0.063 (0.027)	0.080 (0.025)	0.017 (-0.055-0.089)	0.64	0.65	0.075 (0.018-0.132)	**0.010**	**0.016**	0.087 (0.032-0.142)	**1.98E-03**	**2.64E-03**
Smoking	0.108 (0.026)	0.066 (0.024)	-0.045 (-0.116-0.027)	0.22	0.29	0.111 (0.054-0.168)	**1.50E-04**	**3.59E-04**	0.068 (0.014-0.123)	**0.014**	**0.016**
Alzheimer's Disease	-0.025 (0.025)	0.059 (0.025)	0.083 (0.013-0.154)	**0.020**	0.060	-0.024 (-0.081-0.033)	0.41	0.44	0.061 (0.006-0.116)	**0.030**	**0.030**

Notes. All tests were adjusted for sex, age at death (for suicide cohorts), and 20 genetic ancestry principal components. Results in bold type are significant. FDR was calculated within each cohort test (SD-N vs. SD-S; SD-N vs. control; SD-S vs. control). All PGS were normalized to the controls, giving the controls mean PGS of 0 and standard deviation of 1.
SE = standard error of the mean. SD-N = suicide deaths with no evidence of prior nonfatal suicidality. SD-S = suicide deaths with evidence of prior nonfatal suicidality. MDD = major depressive disorder. PTSD = post-traumatic stress disorder. ADHD = attention deficit hyperactivity disorder. FDR-adj = adjusted for False Discovery Rate.

© 2025 Coon H et al. *JAMA Network Open*.

eTable 16. Polygenic score comparisons including all suicides; combined males and females with no ancestry threshold

Polygenic score for trait or diagnosis	SD-N, mean PGS (SE)	SD-S, mean PGS (SE)	SD-N vs. SD-S, adj. mean difference (95% CI) (95% CI)	p-value	FDR adj. p-value	SD-N vs. Controls, adj. mean difference (95% CI) (95% CI)	p-value	FDR adj. p-value	SD-S vs. controls, adj. mean difference (95% CI) (95% CI)	p-value	FDR adj. p-value
Bipolar Disorder	0.108 (0.020)	0.169 (0.020)	0.061 (0.004-0.118)	**0.036**	0.072	0.114 (0.062-0.166)	**1.94E-05**	**7.75E-05**	0.175 (0.124-0.225)	**9.42E-12**	**3.77E-11**
MDD	0.126 (0.022)	0.214 (0.022)	0.081 (0.019-0.142)	**0.010**	0.053	0.136 (0.084-0.189)	**3.36E-07**	**2.02E-06**	0.221 (0.171-0.271)	**8.85E-18**	**5.31E-17**
Depressed Affect	0.038 (0.025)	0.103 (0.023)	0.070 (0.002-0.138)	**0.044**	0.075	0.038 (-0.014-0.091)	0.15	0.18	0.104 (0.053-0.155)	**5.70E-05**	**1.14E-04**
Anxiety	0.081 (0.023)	0.174 (0.023)	0.092 (0.027-0.157)	**5.32E-03**	0.053	0.079 (0.026-0.131)	**3.33E-03**	**5.71E-03**	0.172 (0.122-0.223)	**2.37E-11**	**7.11E-11**
PTSD	0.123 (0.022)	0.179 (0.023)	0.062 (-0.001-0.125)	**0.055**	0.082	0.108 (0.055-0.160)	**5.75E-05**	**1.38E-04**	0.168 (0.118-0.219)	**6.33E-11**	**1.52E-10**
Neuroticism	0.009 (0.023)	0.088 (0.022)	0.078 (0.014-0.142)	**0.017**	0.053	0.007 (-0.046-0.059)	0.80	0.80	0.088 (0.037-0.138)	**6.42E-04**	**1.10E-03**
Autism	0.060 (0.025)	0.088 (0.024)	0.029 (-0.039-0.097)	0.41	0.45	0.060 (0.008-0.113)	**0.025**	**0.037**	0.086 (0.036-0.137)	**8.55E-04**	**1.28E-03**
ADHD	0.100 (0.025)	0.055 (0.024)	-0.045 (-0.112-0.023)	0.20	0.26	0.107 (0.054-0.160)	**7.08E-05**	**1.42E-04**	0.060 (0.009-0.111)	**0.020**	**0.021**
Schizophrenia	0.248 (0.021)	0.319 (0.021)	0.072 (0.013-0.132)	**0.018**	0.053	0.237 (0.185-0.289)	**7.32E-19**	**8.79E-18**	0.313 (0.263-0.363)	**4.55E-34**	**5.46E-33**
Alcohol	0.046 (0.023)	0.066 (0.022)	0.018 (-0.046-0.082)	0.57	0.57	0.058 (0.006-0.111)	**0.030**	**0.040**	0.074 (0.023-0.124)	**4.26E-03**	**5.68E-03**
Smoking	0.106 (0.023)	0.070 (0.022)	-0.039 (-0.102-0.025)	0.24	0.28	0.109 (0.056-0.161)	**5.27E-05**	**1.38E-04**	0.071 (0.021-0.122)	**5.48E-03**	**6.57E-03**
Alzheimer's Disease	-0.014 (0.022)	0.057 (0.022)	0.069 (0.007-0.131)	**0.029**	0.070	-0.013 (-0.066-0.039)	0.62	0.68	0.059 (0.009-0.110)	**0.021**	**0.021**

Notes. All tests were adjusted for sex, age at death (for suicide cohorts), and 20 genetic ancestry principal components. Results in bold type are significant. FDR was calculated within each cohort test (SD-N vs. SD-S; SD-N vs. control; SD-S vs. control). All PGS were normalized to the controls, giving the controls mean PGS of 0 and standard deviation of 1.
SE = standard error of the mean. SD-N = suicide deaths with no evidence of prior nonfatal suicidality. SD-S = suicide deaths with evidence of prior nonfatal suicidality. MDD = major depressive disorder. PTSD = post-traumatic stress disorder. ADHD = attention deficit hyperactivity disorder. FDR-adj = adjusted for False Discovery Rate.

© 2025 Coon H et al. *JAMA Network Open*.

eTable 17. Comparisons of PGS derived using PRS-CS (suicides are restricted to 80% European), combined males and females

Polygenic score for trait or diagnosis	SD-N, mean PGS (SE)	SD-S, mean PGS (SE)	SD-N vs. SD-S, adj. mean difference (95% CI) (95% CI)	p-value	FDR adj. p-value	SD-N vs. Controls, adj. mean difference (95% CI) (95% CI)	p-value	FDR adj. p-value	SD-S vs. controls, adj. mean difference (95% CI) (95% CI)	p-value	FDR adj. p-value
Bipolar Disorder	0.132 (0.025)	0.214 (0.025)	0.072 (0.001-0.143)	**0.046**	0.079	0.137 (0.081-0.193)	**1.66E-06**	**6.64E-06**	0.218 (0.164-0.272)	**2.20E-15**	**8.80E-15**
MDD	0.173 (0.026)	0.300 (0.025)	0.115 (0.044-0.187)	**1.63E-03**	**0.020**	0.182 (0.126-0.239)	**2.23E-10**	**1.34E-09**	0.307 (0.253-0.360)	**7.05E-29**	**4.23E-28**
Depressed Affect	0.061 (0.027)	0.146 (0.026)	0.082 (0.009-0.156)	**0.029**	0.060	0.061 (0.005-0.118)	**0.033**	**0.050**	0.146 (0.092-0.200)	**1.12E-07**	**1.92E-07**
Anxiety	0.025 (0.027)	0.147 (0.026)	0.111 (0.037-0.184)	**3.30E-03**	**0.020**	0.031 (-0.026-0.087)	0.28	0.31	0.151 (0.097-0.205)	**4.02E-08**	**9.65E-08**
PTSD	0.076 (0.027)	0.167 (0.026)	0.083 (0.008-0.158)	**0.030**	0.060	0.074 (0.018-0.130)	**0.010**	**0.020**	0.165 (0.111-0.219)	**2.32E-09**	**6.95E-09**
Neuroticism	0.045 (0.028)	0.152 (0.026)	0.101 (0.027-0.176)	**7.89E-03**	**0.032**	0.036 (-0.020-0.093)	0.21	0.25	0.148 (0.094-0.202)	**8.10E-08**	**1.62E-07**
Autism	0.029 (0.028)	0.063 (0.028)	0.022 (-0.056-0.100)	0.58	0.58	0.036 (-0.020-0.093)	0.21	0.25	0.065 (0.011-0.120)	**1.81E-02**	**1.97E-02**
ADHD	0.100 (0.027)	0.089 (0.027)	-0.024 (-0.100-0.052)	0.53	0.58	0.112 (0.056-0.169)	**9.94E-05**	**2.98E-04**	0.097 (0.043-0.151)	**4.50E-04**	**6.00E-04**
Schizophrenia	0.229 (0.025)	0.329 (0.026)	0.094 (0.022-0.166)	**0.011**	**0.032**	0.227 (0.171-0.283)	**2.15E-15**	**2.58E-14**	0.329 (0.275-0.383)	**8.73E-33**	**1.05E-31**
Alcohol	0.048 (0.027)	0.107 (0.026)	0.059 (-0.016-0.134)	0.12	0.16	0.065 (0.009-0.121)	**0.024**	**0.041**	0.117 (0.063-0.171)	**2.02E-05**	**3.03E-05**
Smoking	0.093 (0.027)	0.078 (0.026)	-0.023 (-0.097-0.052)	0.55	0.58	0.101 (0.044-0.157)	**4.56E-04**	**1.10E-03**	0.084 (0.030-0.138)	**2.36E-03**	**2.84E-03**
Alzheimer's Disease	-0.022 (0.026)	0.050 (0.026)	0.068 (-0.006-0.142)	0.07	0.11	-0.021 (-0.077-0.036)	0.48	0.48	0.050 (-0.004-0.104)	0.07	0.07

Notes. All tests were adjusted for sex, age at death (for suicide cohorts), and 20 genetic ancestry principal components. Results in bold type are significant. FDR was calculated within each cohort test (SD-N vs. SD-S; SD-N vs. control; SD-S vs. control). All PGS were normalized to the controls, giving the controls mean PGS of 0 and standard deviation of 1.
SE = standard error of the mean. SD-N = suicide deaths with no evidence of prior nonfatal suicidality. SD-S = suicide deaths with evidence of prior nonfatal suicidality. MDD = major depressive disorder. PTSD = post-traumatic stress disorder. ADHD = attention deficit hyperactivity disorder. FDR-adj = adjusted for False Discovery Rate.

© 2025 Coon H et al. *JAMA Network Open*.
