# Supplementary material for: Genetic Liabilities to Neuropsychiatric Conditions in Suicide Deaths With No Prior Suicidality
Source: JAMA Netw Open. 2025 Oct 20;8(10):e2538204. doi: 10.1001/jamanetworkopen.2025.38204 (PMC12538367; doi:10.1001/jamanetworkopen.2025.38204)
Supplement: Supplement 2. — Data Sharing Statement [file jamanetwopen-e2538204-s002.pdf]

Data Sharing Statement

Coon. Genetic Liabilities to Neuropsychiatric Conditions in Suicide Deaths With No Prior Suicidality. *JAMA Netw Open*. Published October 20, 2025.
doi:10.1001/jamanetworkopen.2025.38204

Data
Data available: Yes
Data types: Deidentified participant data
How to access data: Data will be available upon request from the corresponding author, and with the permissions required by institutional regulations and state statutes
When available: With publication

Supporting Documents
Document types: Other (please specify)
Additional Information: Summary of results from prior clinical studies in these cohorts (eTable 1-eTable 3); comparison of study data freeze to full USMRS data cohort (eTable 4); summary of NLP validation and deployment (eTable 5-eTable 10); details of genetic imputation and QC (eAppendix); source studies from which PGS were computed (eTable 11); intercorrelations of PGS in this study (eTable 12); distributions of age at death for suicide cohorts (eFigure); polygenic score results between SD-N vs. SD-S unadjusted for age and sex to allow inspection of effects of these two significant moderators (eTable 13); polygenic score results between SD-N vs. SD-S within subsets defined by sex and by age at death (eTable 14); polygenic score results between SD-N vs. SD-S using a more restrictive ancestry threshold of 90% EUR (eTable 15); polygenic score results between SD-N vs. SD-S using no ancestry threshold (eTable 16); polygenic score restults between SD-N vs. SD-S using PGS computed with PRS-CS to allow inspection of robustness to method of PGS scoring (eTable 17)
How to access documents: uploaded with manuscript
When available: With publication

Additional Information
Who can access the data: data from suicide deaths described in the manuscript will be available upon request from the corresponding author, and with the permissions required by institutional regulations and state statutes; the supplementary table will be uploaded with the manuscript
Types of analyses: analyses using the data from suicide deaths must not be for commercial purposes, and must first be approved according to institutional regulations and state statutes
Mechanisms of data availability: analyses using the data from suicide deaths must not be for commercial purposes, and must first be approved according to institutional regulations and state statutes
Any additional restrictions: analyses using the data from suicide deaths must not be for commercial purposes, and must first be approved according to institutional regulations and state statutes
